# Global Literature Analysis of Tumor Organoid and Tumor-on-Chip Research

**DOI:** 10.3390/cancers17010108

**Published:** 2025-01-01

**Authors:** Jun-ya Shoji, Richard P. Davis, Christine L. Mummery, Stefan Krauss

**Affiliations:** 1 Hybrid Technology Hub, Centre of Excellence, Institute of Basic Medical Sciences, University of Oslo, 0372 Oslo, Norway; 2 Department of Anatomy & Embryology, Leiden University Medical Center, 2300 RC Leiden, The Netherlands; 3 The Novo Nordisk Foundation Center for Stem Cell Medicine (reNEW), Leiden University Medical Center, 2300 RC Leiden, The Netherlands; 4 Department of Applied Stem Cell Technologies, University of Twente, 7522 NB Enschede, The Netherlands

**Keywords:** tumor organoid, tumor-on-chip platforms, text mining, microphysiological systems

## Abstract

**Background**: Tumor organoid and tumor-on-chip (ToC) platforms replicate aspects of the anatomical and physiological states of tumors. They, therefore, serve as models for investigating tumor microenvironments, metastasis, and immune interactions, especially for precision drug testing. To map the changing research diversity and focus in this field, we performed a quality-controlled text analysis of categorized academic publications and clinical studies. **Methods**: Previously, we collected metadata of academic publications on organoids or organ-on-chip platforms from PubMed, Web of Science, Scopus, EMBASE, and bioRxiv, published between January 2011 and June 2023. Here, we selected documents from this metadata corpus that were computationally determined as relevant to tumor research and analyzed them using an in-house text analysis algorithm. Additionally, we collected and analyzed metadata from ClinicalTrials.gov of clinical studies related to tumor organoids or ToC as of March 2023. **Results and Discussion**: From 3551 academic publications and 139 clinical trials, we identified 55 and 24 tumor classes modeled as tumor organoids and ToC models, respectively. The research was particularly active in neural and hepatic/pancreatic tumor organoids, as well as gastrointestinal, neural, and reproductive ToC models. Comparative analysis with cancer statistics showed that lung, lymphatic, and cervical tumors were under-represented in tumor organoid research. Our findings also illustrate varied research topics, including tumor physiology, therapeutic approaches, immune cell involvement, and analytical techniques. Mapping the research geographically highlighted the focus on colorectal cancer research in the Netherlands, though overall the specific research focus of countries did not reflect regional cancer prevalence. These insights not only map the current research landscape but also indicate potential new directions in tumor model research.

## Simple Summary

Tumor organoids and tumor-on-chip are three-dimensional tumor cultures that are increasingly used to model cancers in preclinical research. To reveal how these emerging technologies are transforming oncological research, we analyzed ~3500 academic publications and 139 clinical trials by using a quality-controlled text-mining algorithm. To date, we found that 55 and 24 tumor classes have been modeled as tumor organoids and tumor-on-chip models, respectively, with notable increases in recent research activity on neural and hepatic/pancreatic tumor organoids. Comparative analysis with cancer statistics showed that lung, lymphatic, and cervical tumors remain under-represented in tumor organoid research. We also explored the research scope with these models, including topics such as tumor physiology, therapeutic approaches, immune cell involvement, and analytical techniques. Finally, mapping the research geographically highlighted the prominence of colorectal cancer research in the Netherlands, although overall, the specific research focus of countries did not reflect regional cancer prevalences.

## 1. Introduction

Globally, cancer is a leading cause of premature death. It was ranked as the top cause in 57 countries in 2019 [1]. The significant health burden of cancer is expected to increase significantly in the next decades, with a projected 47% rise in new cancer cases from 2020 to 2040 [2].

One important challenge in preclinical cancer research has been establishing model systems that reliably recapitulate the patient’s condition [3]. Human models are indispensable for understanding tumor physiology and translating this knowledge into effective cancer treatments. Ideally, a good model would faithfully recreate tumor physiology in vivo, including components and complex aspects that pose major challenges to clinical treatments, such as tumor heterogeneity (i.e., genetic and phenotypic variation among tumors or tumor cells) and the tumor microenvironment (i.e., tissue context of the tumor, as well as the presence of stromal and immune cells, blood vessels, and extracellular matrix) [3,4]. These models must also be robust, reproducible, and cost- and time-efficient, as information on the best treatment for each patient is often urgent. Furthermore, these are cornerstones for high-throughput analysis and drug screening.

Among the commonly used tumor model systems, two-dimensional in vitro cancer cell cultures are highly cost- and time-effective but often fail to recreate complex aspects of tumor physiology [5]. Mouse-based models, including genetically engineered mouse models and tumor xenografts (i.e., tumor tissues grafted to a non-human animal), are in vivo paradigms that support aspects of complex tumor physiology but are expensive and time-consuming. They also have associated ethical concerns for animal welfare and limited human disease predictability [3,6,7]. In contrast, tumor organoids and tumor-on-chip (ToC) systems offer promising alternatives. These in vitro models provide three-dimensional (3D) representations of tumor structures and functionality, are rated intermediate-to-high in terms of cost- and time effectiveness and ability to recreate tumor physiology, and are considered to bridge the gap between simple cell cultures and complex mouse-based models [3,7,8].

Organoids are self-organizing, 3D tissue cultures that model aspects of organ development, composition, and function [9]. Tumor organoids (sometimes referred to as tumoroids or tumoroids) are a type of organoid intended to model aspects of tumor physiology [3,5,7,8]. Following the establishment of protocols for generating non-tumor organoids [10,11], tumor organoids were generated in 2011 from mouse intestinal adenoma and endoscopic biopsy samples of colon cancer patients [12]. Since then, a wide range of tumor organoids have been developed from patient-derived tissue, mouse tumors, cancer cell lines, or stem cells with oncogenic mutations [3,13] to model multiple tumors, including breast [14], pancreatic [15], prostate [16], liver [17], brain [18], lung [19], gastric [20], ovarian [21], and bladder [22]. As a relatively new technology, tumor organoid models are still rapidly developing, for example, in terms of capturing more complex tumor physiology that includes tumor heterogeneity and microenvironment [3]. Nevertheless, tumor organoid models have already proven useful for preclinical cancer research, including basic science discoveries, drug development and screening, personalized or precision medicine, and the establishment of biobanks [8,13].

ToC platforms, which utilize microfluidic devices to control liquid flow through and between cell cultures grown within the chip, are designed to recapitulate parts of the tumor microenvironment [4,8,23]. Similar to tumor organoid models, several ToC platforms have been described in the past decade; these have recently been extensively reviewed [23]. ToC models include lung [24], breast [25], prostate [26], colorectal [27], pancreatic [28], and skin [29] cancers, among others. One research focus of ToC is pharmacology research, including drug development and screening; however, they have also proven useful for basic research on the tumor microenvironment [4,23].

Collectively, tumor organoid and ToC technologies have expanded tremendously over the last decade in terms of types of tumors represented, preclinical applications, and culture and analytical methods [8,13,23]. This is reflected in the rapid increase in the number of related academic publications on organoid and organ-on-chip (OoC) in general [30]. Recently, we performed a literature analysis of the global research on non-tumor organoids and OoC from 2011 onwards, using an in-house purpose-built algorithm [30]. Here, we expanded the algorithm to perform a similar analysis of the global tumor organoid and ToC research, as our approach is better suited for analyzing large numbers of documents in sufficient depth in a reproducible and easily updatable manner, compared to conventional approaches, such as systematic reviews and bibliometric analysis. Our goal is to provide a comprehensive overview of research on these model systems in order to map the changing research diversity and focus in this field and to identify current challenges. Specifically, we investigated which research subareas and topics are increasingly studied and which are still under-researched. Within ~ 3500 academic publications and 139 clinical trials, we first classified tumor organoids and ToC models based on organs and substructures from which they were derived, showing increasing publication output in the past few years on neural and hepatic/pancreatic tumor organoids, as well as gastrointestinal, neural, and reproductive ToC models. Comparative analysis with incidence and mortality of corresponding tumor types showed that lung, lymphatic, and cervical tumors are still under-represented in tumor organoid research; however, signs of increasing research activity are noted. Research topic analysis identified immunotherapy, immune cells, and (sub)cellular tumor physiology as emerging research foci in tumor organoid research. To date, global research has been led by the USA and European Research Area (ERA), while increasing research activities are seen in particular for ToC in a range of countries. Overall, in this study, we provide a comprehensive research landscape of tumor organoid and ToC technologies, with emphasis on recent research trends.

## 2. Materials and Methods

The publication metadata were collected and analyzed as described in detail previously [30], with additional oncological analysis. A summary of the methods is given below. The code used for the analysis is available at the GitHub repository (https://github.com/jyshoji/text_analysis_tumor_organoids (accessed on 20 December 2024); DOI: 10.5281/zenodo.13890271).

### 2.1. Collecting Academic Publications on Organoids and OoCs

Metadata of academic publications on organoids and OoCs (including tumor organoids and ToC) were collected from the following four literature databases.

- EMBASE via Ovid (https://ovidsp.ovid.com);
- PubMed (https://pubmed.ncbi.nlm.nih.gov);
- Scopus (https://www.scopus.com/);
- Web of Science (https://www.webofscience.com/);

Metadata of preprint publications were collected from bioRxiv (https://www.biorxiv.org).

The following two sets of search terms were used to identify academic publications on organoids:

- organoid OR enteroid OR colonoid OR assembloid OR gastruloid OR iblastoid OR tumoroid (including plural forms; 2011 to present);
- (blastoid OR blastoids) AND embryonic (2018 to present).

Some of these search terms are irrelevant to tumor organoids but were nevertheless included, as we first collected publications on any organoids, from which we selected tumor organoid publications.

In order to broadly identify academic publications on OoCs, which use a wide range of nomenclature. First, we identified and retrieved academic publications on the on-chip technology; from these, we subsequently selected OoC publications. We also identified academic publications on microphysiological systems (MPS). Collectively, the following two sets of search terms were used to identify academic publications:

- “on-chip” OR “on-chips” OR “on-a-chip” (2011 to present; note that hyphens were replaced with white space for some databases);
- “microphysiological system*” OR “microphysiology system*” OR “microphysiologic system*” OR “micro physiological system*” OR “micro physiology system*” OR “micro physiologic system*” (where * works as a wildcard character; 2011 to present).

We only collected metadata of academic publications that were written in English and were classified in the databases as “article” and/or “review” while excluding “comment”, “editorial”, “retracted article”, and “retraction notice” when possible. Hereafter, each non-review academic publication collected is referred to as a “research article”. Notably, as we did not include non-English publications, our analysis may have missed some regional research activities and trends. The literature search and collection were initiated on 6 September, 2022 and updated on 6 June 2023 by adding publications published after 1 January, 2022 and filtering out duplicate publications. The nine-month overlap of publication periods (January to September, 2022) between the two data acquisitions was introduced to handle changes in the publication year, which tend to occur when publication status changes from electronic to printed publication. Accordingly, if a publication in the existing corpus was also present in the newly added corpus, the publication year was changed according to the latter, before the new copy was discarded as a duplicate document.

The metadata, including titles and abstracts of the identified publications, were exported as either .ris (EMBASE), .nbib (PubMed), or .bib (Scopus, Web of Science, and bioRxiv) file formats using the export function of each database. In metadata files originating from PubMed or Scopus, all occurrences of em- and en-dash were replaced with a standard dash, and non-breaking spaces were replaced with standard spaces as these characters would have been skipped when the files were imported to R (R core team, 2021).

### 2.2. Metadata Cleanup and Deduplication

Analyses were performed using R [31] (version 4.0.5) on RStudio [32] (version 2023.03.1+446) using the tidyverse package [33]. The complete code used for this section can be found at/R/formatting.R of the repository and can be opened and run on RStudio.

The metadata corpora were loaded on R and deduplicated (i.e., removing extra copies of the same document) using the revtools package [34], as described previously [30]. From the on-chip corpus, academic publications were selected where the terms “on-chip”, “on-a-chip”, or “on-chips” followed organ names or organotypic processes (e.g., angiogenesis). The resulting academic publications were combined with the MPS corpus to give the integrated OoC corpus, which was further deduplicated.

### 2.3. Identification of Tumor Models

Organ types of origin for organoid or OoC models were determined in each document primarily according to the organ names that appeared before the term organoid (for documents in the organoid corpus) or on-chip/on-a-chip/on-chips (in OoC corpus). When this step failed, the organ type was instead determined according to the organ names that appeared in sentences that included the terms organoid/on-chip/on-a-chip/on-chips. When oncological terms were identified together with organ names in the above steps, the corresponding document was assigned to either the “tumor organoid” or “ToC” corpus. The current paper reports an analysis of the tumor organoid and ToC corpora, whereas the analysis of the remaining corpora was published elsewhere [30].

The organ types determined above in tumor organoid and ToC corpora still included non-tumor models, as some publications used both non-tumor and tumor models. Thus, we further determined tumor organ models by selecting those matching researched tumor types in each publication. For validation, we manually classified tumor models in three subsets of documents in the corpora (n = 24 for each) and compared them with the algorithm-based classification, which showed 89 ± 5% matches (mean ± SD), indicating an approximate 10% misclassification rate. Considering this misclassification rate, we mostly focused on differences in numbers that are bigger than this value; for example, we considered >40% changes in publication counts as significant upward/downward trends.

### 2.4. Identifying Countries/Regions of Authors’ Affiliations

To determine the countries of origin for the research, country/region names were identified in the author address field. Next, the number of times each country/region name appeared was counted for each document. The country/region with the highest number of occurrences was determined in each document to be the main research country. Where more than one country/region was found with the same number of occurrences, the country/region mentioned earliest was chosen, which typically represented the first author’s country. Research articles from one of the countries affiliated with the European Research Area (ERA) were also grouped and summarized as an additional pie chart in global research trends. ERA here was defined as the participants of the Horizon 2000/Europe, consisting of 27 EU member states (including the former member UK) and 18 associated countries (including the former member Switzerland).

Fractional counts of each country’s contributions were also calculated for each document as defined by the number of occurrences of a country/region name divided by the total number of occurrences of all country/region names. For regional research trends, the fractional counts of contributions were further adjusted by the country’s population, which was taken from Wikipedia [35].

### 2.5. Identifying Research Topics

A nested list of research topics (e.g., clinical and oncological terms) was made, in which the second-level lists contained various writing styles and synonyms of the corresponding first-level lists. The terms in the second-level lists were captured in titles, keywords, and abstracts, and summarized according to the first-level lists.

### 2.6. Collecting and Analyzing Metadata of Clinical Trials

Metadata of clinical trials using the term tumor organoids and/or ToC were obtained from ClinicalTrials.gov (https://clinicaltrials.gov) on 22 March 2023. The search conditions were as follows: Status: *All studies*; Condition or disease: *Tumor*, with the following two search strings used for Other terms to separately identify clinical trials using tumor organoids and ToC.

Tumor organoids: organoid OR organoids OR enteroid OR enteroids OR colonoid OR colonoids OR tumoroid OR tumoroids OR tumouroid OR tumouroids.

ToC: on-chip OR on-chips OR on-a-chip.

Excluding withdrawn trials, we identified and retrieved metadata of 139 clinical trials collectively, which we loaded and analyzed on RStudio. Primary purposes of clinical trials were identified in the Study Designs column of the metadata. Researched tumor types were identified in the Conditions column using the code snippets used for identifying organ models in academic publications so that tumor types are consistently grouped.

### 2.7. Graphical Visualization of the Results

The circular packing graph and diagrams were drawn using the igraph (version 1.5.1) [36], ggpp (version 0.5.8-1) [37], and ggraph (version 2.1.0) [38] packages. World maps showing global research trends were drawn using the rworldmap package (version 1.3-8) [39]. The R Graph Gallery [40] was referred to for inspiration for visualization methods.

## 3. Results and Discussion

### 3.1. Overview of Academic Publications on Tumor Organoids and ToC

Academic publications on organoids and OoCs from 2011 onwards were collected from four major academic publication databases (EMBASE, PubMed, Scopus, Web of Science), as well as the preprint database bioRxiv. Each publication was assigned to either “organoid”, “tumor organoid”, “OoC”, or “ToC” corpora through text analysis [30]. For this analysis, we focused on the “tumor organoid” and “ToC” corpora. The text analysis step selected publications that included terms representing tumors and 3D culture models in close proximity to each other (within either four words or the same sentence, depending on context), with the purpose of excluding publications that merely mention tumor (e.g., as part of future directions), such that the “tumor organoid” and “ToC” corpora mostly consisted of publications on 3D tumor models. Hereafter, we use the term “research articles” to refer to non-review publications and “academic publications” to refer to both non-review and review publications. The “review” and “non-review” classifications were predetermined in the publishers/literature databases before retrieval, meaning that “research articles” in our corpora still include opinion articles. While our approach is based on computationally reading titles, keywords, and abstracts of academic publications, with manual adjustments being performed where necessary. For example, computational classifications were manually checked and corrected for infrequently studied organ representations of tumor models.

In total, our tumor organoid corpus consisted of 3066 academic publications encompassing 2171 research articles, 627 reviews, and 268 preprints, while the ToC corpus consisted of 485 academic publications encompassing 286 research articles, 189 reviews, and 10 preprints. A total of 15 research articles, 54 reviews, and one preprint were present in both corpora, as they mentioned both tumor organoids and ToC. In the subsequent analysis, preprint publications were excluded because of their small numbers and the fact that they were not peer-reviewed. From 2011 to 2022, the number of research articles on tumor organoids and ToC saw average annual growth of 45% and 59%, respectively. As such, by 2022, research articles using tumor organoid and ToC models comprised 0.33% and 0.032% of all tumor research articles, respectively. The yearly publication counts of tumor organoid research and tumor research could be fitted to the following exponential regression equations, where *x* is a year and *y* is the number of research articles (either on tumor organoid or in tumor research as a whole) in that year:

ln(*y*) = −808.74507 + 0.40315 ∗ *x*, (adjusted *r*^2^ = 0.9806, F-statistic = 558.1, *p* = 4.189 × 10^−10^) (tumor organoid research);

ln(*y*) = −93.706799 + 0.052309 ∗ *x*, (adjusted *r*^2^ = 0.9734, F-statistic: 404.3, *p* = 2.036 × 10^−9^) (tumor research as a whole).

Based on current growth, these regression models predict that tumor organoids may be used as a model in 1% of total tumor research articles by 2025 and in 5% of articles by 2030.

As of March 2023, the ClinicalTrials.gov (https://clinicaltrials.gov) database listed 139 clinical trials related to tumor studies using organoids/OoC technology, not including withdrawn trials. Of these, 135 trials mentioned organoids, five mentioned OoC, and one trial referenced both technologies. Most of these trials focus on collecting biopsy samples to develop tumor organoid models for disease modeling and/or drug testing, which later may be translated into precision medicine. The number of studies registered started showing steady growth in 2014, with “treatment” becoming the major primary purpose of trials in recent years (Figure S1).

### 3.2. Tumor Organoid Models and Their Trends

**Figure 1:**
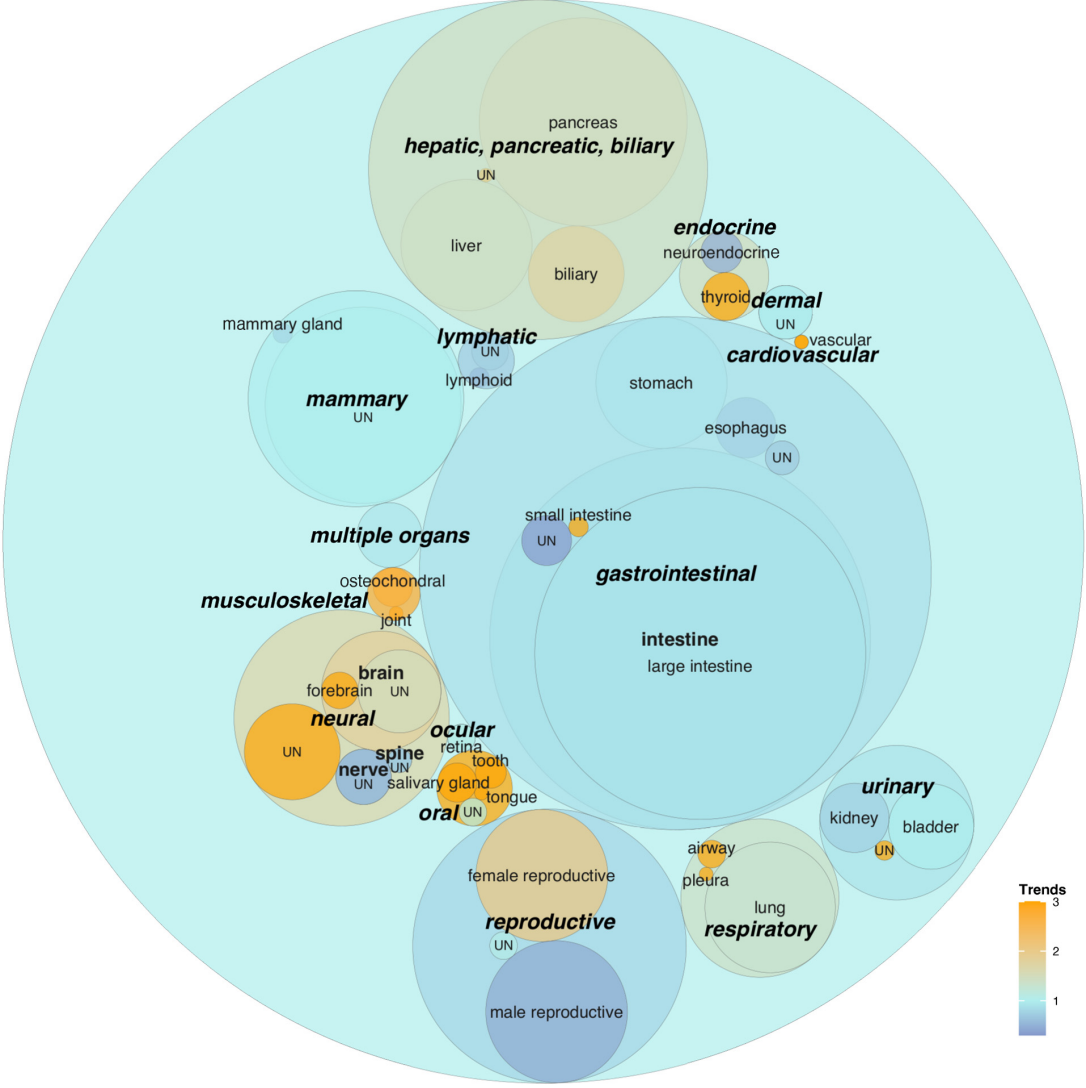
Publication trends of organs and substructures modeled by tumor organoids. Circular packing graph showing two-level hierarchical classifications of organs and substructures of origin modeled by tumor organoids. Only research articles in which tumor organoid models were computationally determined were included in the plot (1839 research articles out of 3066 academic publications). Gastrointestinal and neural models include an extra level of categories. Sphere size reflects the number of research articles on the corresponding organ model. The color shows publication trends as calculated by relative increases in the number of research articles in recent years (from 2020 onwards) compared to the earlier period (2011–2019 inclusive) for each organ/substructure of origin and then adjusted for the relative increase in the entire corpus. “UN” (i.e., unspecified) as a category represents papers classified as studying the corresponding upper-level organoid category without a computer-recognizable description of the specific lower-level categories. The “neuroendocrine” category under “endocrine” was only used when it was the only category that the algorithm detected in a publication. When another organ category was detected, the publication was categorized according to this other organ (e.g., “neuroendocrine prostate cancer” was categorized as “prostate”, instead of “neuroendocrine” or “multiple organs”). Note that spheres representing neural and gastrointestinal categories are relatively enlarged due to housing an extra level of lower categories. See Figure S2 for the number of publications included in each category.

**Figure 2:**
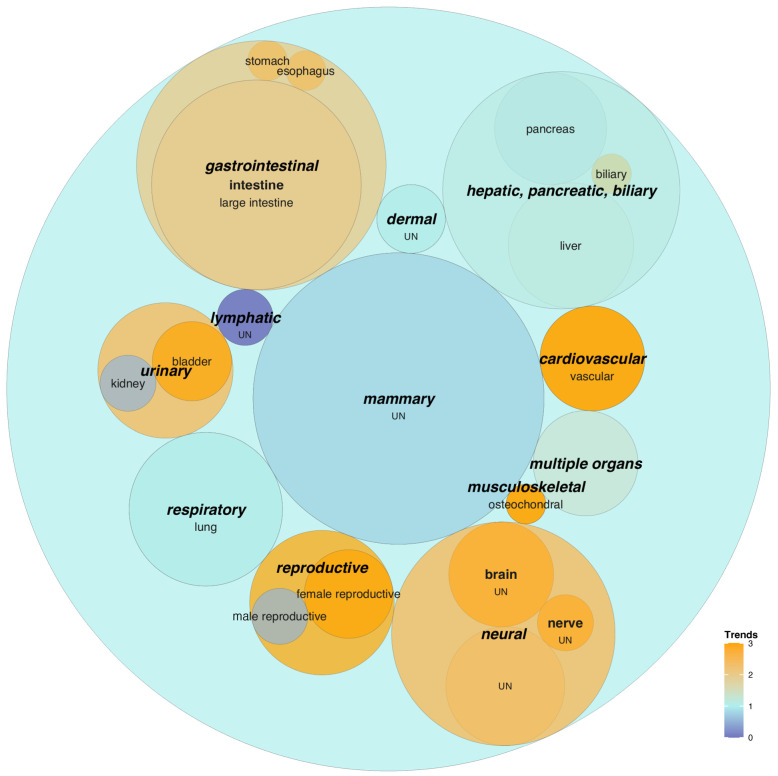
Publication trends of organs and substructures modeled by ToC. Circular packing graph showing two-level hierarchical classifications of organs and substructures of origin modeled by ToC. Only research articles in which researched ToC models were computationally determined were included in the plot (169 research articles out of 485 academic publications). See Figure S3 for the number of publications included in each category.

Following the nomenclature used by the authors in research articles, we identified 55 and 24 tumor classes modeled as tumor organoids and ToC, respectively. These were classified into hierarchical categories (Figures S2 and S3, Tables S1 and S2) based on anatomical structures and substructures, with adaptations to accommodate previously published organoid classifications [9,30,41]. Figure 1 (tumor organoids) and Figure 2 (ToC) show the first and second levels of the hierarchical organ/substructure categories that were identified by the classification, including an extra level for neural and gastrointestinal organs.

Gastrointestinal tumor organoids are the research subarea with the highest publication count, primarily due to the high research activity on large intestine models (Figure 1). This parallels a high research activity on the corresponding non-tumor organoid models [30] and reflects a considerable research interest in colorectal cancer. Gastrointestinal tumor organoids also encompass emerging models of the rectum, small intestine, and gastric antrum (Figure S2). The upward trend of rectal tumor organoids (15 research articles since 2020) appears driven by research addressing locally advanced rectal cancer (6 research articles) and/or neoadjuvant therapy (i.e., preliminary cancer therapy that is administrated before the main therapy; 8 research articles). The newly reported small intestine and gastric antrum tumor organoids were used to address tumorigenesis in mouse small intestinal adenoma [42] and human gastric cancer [43], respectively.

Hepatic, pancreatic, and biliary (HPB) tumor organoids comprise the second most studied subarea, with an upward trend in the publication count (Figure 1). Pancreatic tumor organoids represent the largest subcategory, with the upward trend driven by the growing number of publications modeling pancreatic ductal adenocarcinoma. The predominant models for liver tumors are hepatocellular carcinoma, while biliary tumor organoids are also frequently used for modeling cholangiocarcinoma [44], with a strong increase in recent publication counts. Additionally, this group includes uncommon and rarer cancers, such as gallbladder cancer and pancreatic neuroendocrine tumor, which are studied using gallbladder [45] and islet tumor organoids [46], respectively (Figure S2).

Mammary (gland) and reproductive tumor organoids rank as the third and fourth most studied models (Figure 1). Although prostate cancer was the focus of earlier research on reproductive system tumor organoids, publications on tumors of the female reproductive system have increased notably in the past three years, in particular for ovarian cancer (Figure S2). Neural tumor organoids are the fifth most studied and include brain models with an upward publication trend (Figure 1) to study glioma and glioblastoma [47]. Brain tumor organoids also include an emerging model of the pituitary organ (Figure S2) to study pituitary tumors [48].

Other tumor organoids (Figure S2) include a mouse tongue model of oral squamous cell carcinoma [49], patient-derived models of papillary thyroid cancer [50], non-involuting congenital hemangiomas [51], synovial sarcoma [52], salivary gland tumors [53], nasopharyngeal carcinoma [54], a tooth model of ameloblastoma [55], human cell line models of hypopharyngeal cancer [56], and bone models of osteosarcoma [57] and chondrosarcoma [58].

### 3.3. ToC Platforms and Their Trends

The 286 research articles on ToC as of June 2023 were about 13% of the size of the tumor organoid corpus. As a comparison, the number of research articles on OoC was 30% of the whole corpus of non-tumor organoids [30]. Thus, ToC research appears still at an early phase of research development. In addition, the algorithm was unable to classify the organ type for 41% of research articles on ToC (i.e., “unidentified” category in Figure S3), as compared to 15% in the tumor organoid corpus (Figure S2). This is because a large fraction of ToC research articles concern either the development/optimization of microfluidic platforms or (multi)cell-level physiology. Nevertheless, a range of organ representations have been studied as ToC with an overall upward publication trend.

Mammary (gland) tumor models represent the most researched ToC platforms (Figure 2). These are followed by gastrointestinal models with an upward publication trend that encompass newly developed ToC models of the esophagus and stomach (Figure S3) for studying esophageal carcinoma [59] and diffuse-type gastric cancer [60]. HPB models are the third most studied and include a recently developed 3D-bioprinted cholangiocarcinoma model [61]. This is followed by neural models with a rapidly increasing publication trend reflecting growing interest in modeling glioma [62], glioblastoma [63], and neuroblastoma [64]. Other emerging ToC models include those of blood to study leukemia [65], bone for osteosarcoma [66], ovary [67], and bladder [68] for corresponding cancers. Additionally, cervical ToC models are being explored to measure the tropism of stromal cells toward cancer cells.

### 3.4. Comparison of Cancer Statistics, Academic Research, and Clinical Trials Using 3D Culture Model Systems

**Figure 3:**
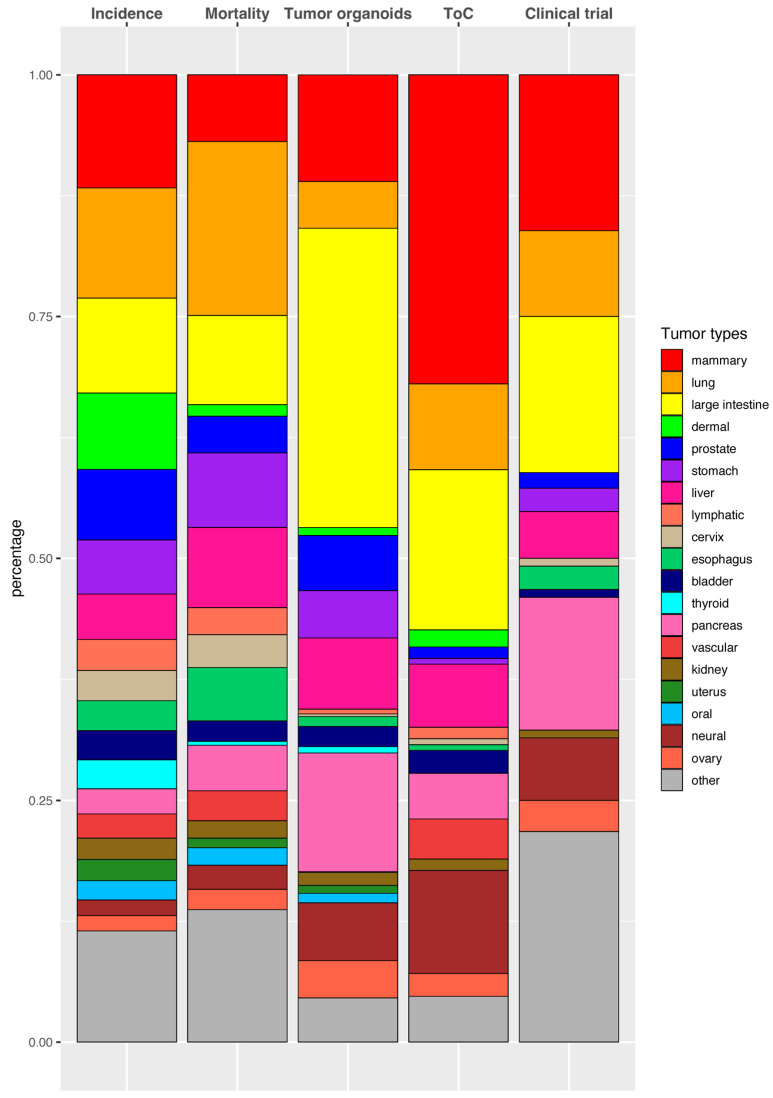
Global incidence, mortality, model systems, and clinical trials by tumor types. Bar chart showing proportions of tumor types in global incidence, global mortality, tumor organoid and ToC research articles, and clinical trials. The proportions of global incidence and mortality were calculated based on Sung et al. [2]. In regard to research articles and clinical trials, only those with computer-determined organ types were included (1839 and 169 research articles for tumor organoids and ToC, respectively; 124 clinical trials). Note that the tumor type grouping is slightly different from that in previous figures in order to be comparable with Sung et al. For example, cholangiocarcinoma is included in the “liver” group here rather than “biliary”.

Figure 3 compares the proportions of tumor types in terms of global incidence and mortality [2] with the number of research articles using tumor organoids and ToC, along with the studies registered on ClinicalTrials.gov.

Tumor organoid research aligns well with the global incidence and mortality rates for mammary, prostate, stomach, and liver tumors. However, large intestinal, pancreatic, and neural tumor organoids have been disproportionately studied. In part, the over-representation of pancreatic and neural tumor organoids could be due to the very high mortality rates associated with these tumors (e.g., pancreatic cancer: 5-year relative survival rate of 12% when all stages are combined [69]), and hence, an urgent clinical need to find predictive models. In contrast, lung, lymphatic, and cervical tumors are under-represented in tumor organoid research, although lung tumor organoids show signs of increasing research output (Figure 1 and Figure S2). In addition, a detailed protocol was recently reported for culturing cervical tumor organoids [70], which may facilitate future research on this under-represented tumor. Taken together, ongoing scientific efforts mitigate the disparity between incidence/mortality and tumor organoid research. Of note, the disparity may also reflect the utility of tumor organoid technology in studying rare cancers with limited sample availability [71].

In ToC research, mammary and neural tumors are over-represented, while prostate and stomach tumors are under-represented. As most registered clinical trial studies use tumor organoids rather than ToC, the tumor groups in these studies appear to mirror tumor organoid models used in the research, with large intestinal, mammary, and pancreatic tumors most represented. Overall, the number of research articles on ToC and clinical trials using tumor organoids/ToC are too low to reflect global incidence/mortality correctly; whereas we noted signs that tumor organoid research is now beginning to reflect global tumor statistics better.

### 3.5. Research Topics in Tumor Organoid Research

**Figure 4:**
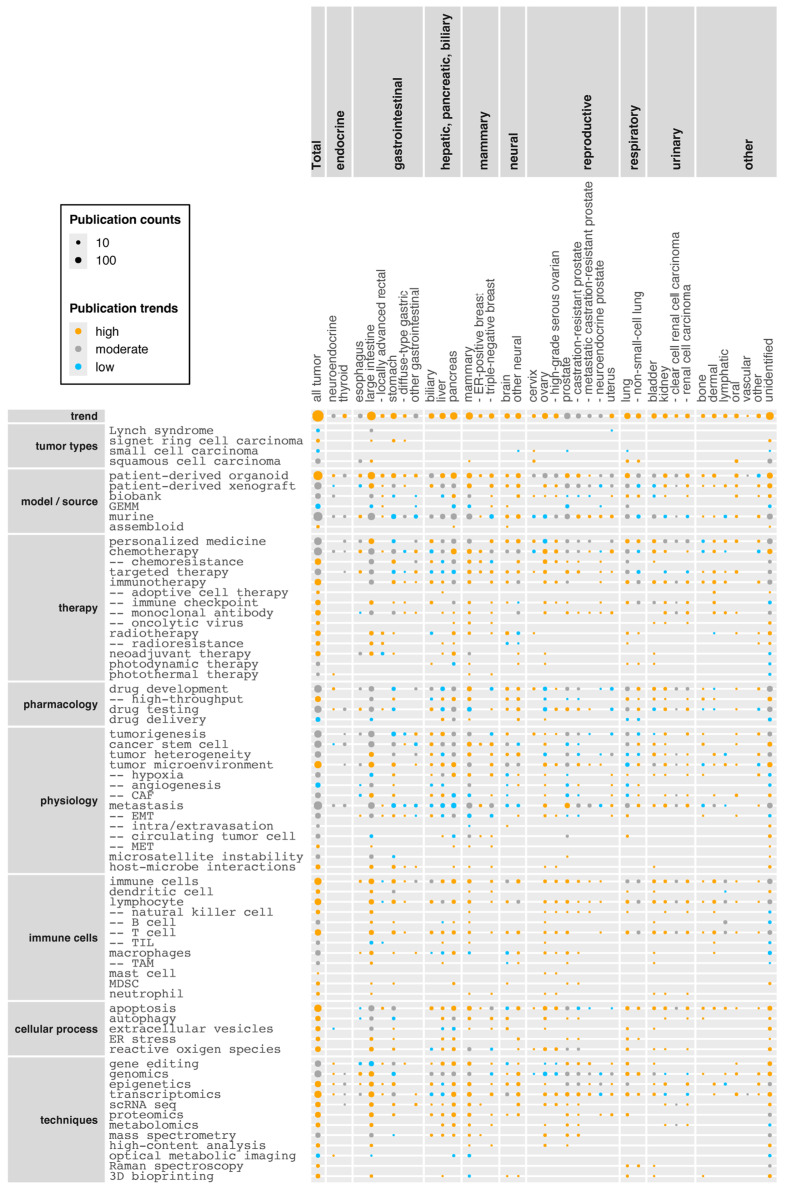
Trends of research topics in tumor organoid research. The correlation matrix shows a correlation between researched tumor groups (on the *x*-axis) and selected research topics (on the *y*-axis), along with their publication trends in tumor organoid research. The size of the spheres reflects the number of research articles in the tumor groups that mention the respective research topics. The color of the spheres reflects trends of the publication counts (upward trends in orange, downward trends in blue). The top row “trend” shows the relative increases in publication counts in recent years (from 2020 onwards) compared to the earlier period (2011–2019) without adjustments. For all the other rows, trends were adjusted in each column, calculated by relative increases in the number of research articles on combinations of tumor types and research topics, which were then divided by the relative increases in publication counts of the corresponding tumor types. In either case, >40% relative increases were considered “high”, whereas >40% relative decreases were considered “low”. Values in between were classified as “moderate”. The *x*-axis category “unidentified” encompasses research articles where the algorithm did not detect researched tumor types. The research topic categories in the *y*-axis represent groups of synonyms and similar terms. For example, “personalized medicine” includes precision medicine, personalised medicine, and other terms. Abbreviation in *x*-axis: ER-positive breast: estrogen-receptor-positive breast cancer. Abbreviations in *y*-axis: GEMM: genetically engineered mouse model, CAF: cancer-associated fibroblast, EMT: epithelial-mesenchymal transition, MET: mesenchymal-epithelial transition, TIL: tumor-infiltrating lymphocyte, TAM: tumor-associated macrophage, MDSC: myeloid-derived suppressor cell, ER stress: endoplasmic reticulum stress, scRNA seq: single-cell RNA sequencing.

Figure 4 shows trends in thematic topics within tumor organoid research (see Table S3 for the list of research articles). Note that the trends in the top row “trend” are unadjusted and show fold changes in the number of research articles on groups of tumors (*x*-axis) between the early (2011–2019) and later (2020–present) phases. The other rows’ present trends were adjusted within columns for the total publication trend of corresponding tumor groups. Furthermore, >40% relative increases were considered as “high”, >40% relative decreases as “low”, and values in between were classified as “moderate”.

Most tumor groups are increasingly being studied using tumor organoid models, with a few exceptions (e.g., prostate cancer) (Figure 4). Notably, a small number of research articles have examined uncommon or rare types of tumors and related conditions (“tumor types” group in the *y*-axis), including “Lynch syndrome” and “signet ring cell carcinoma” in the large intestine and stomach, as well as “small cell carcinoma” in cervix, prostate, and lung.

Tumor organoid models are increasingly generated from patient-derived material (“patient-derived organoid”) across the majority of tumor groups. This is because tumor organoid technology provides an approach for personalized in vitro analysis that does not rely on cancer cell lines, allowing recreation of both genomic and histological characteristics of tumors of individual patients. Additionally, patient-derived tumor organoids can be used to capture tumor heterogeneity within individual patients [72], for example, among different regions of a tumor or between primary and metastatic tumors [73] or recurrent tumors [21]. The use of patient-derived organoids is particularly high in biliary tumor research (Figure S4), where establishing cholangiocarcinoma cell lines is difficult [74] and routine live extraction methods of tumor cells are present. Similarly, “patient-derived xenograft” models are increasingly mentioned across multiple tumor groups (Figure 4), reflecting the use of such xenografts as complementary in vivo models to tumor organoid-based in vitro analysis. For example, patient-derived xenografts are frequently mentioned in prostate cancer research (Figure S4) as xenografts are extensively used as a source for establishing tumor organoids due to the low success rate from biopsy specimens [75,76]. Similarly, patient-derived xenografts may also be used as a source for developing tumor organoids from rare cell sources such as circulating tumor cells [77]. Conversely, tumor organoids may be used as a source for establishing xenografts from rare cancers such as epithelioid sarcoma [78].

Although a “biobank” (i.e., a repository of biological samples) can be a source of cells for generating (tumor) organoids/OoC, the term, when appearing in abstracts, mostly refers to establishing biobanks rather than their use. Tumor organoid biobanks have been established from a wide range of tumors (Figure 4) [79], including less common or rare cancers such as neuroendocrine carcinoma [80], intraductal papillary mucinous neoplasm [81], glioblastoma [82], nasopharyngeal carcinoma [83], and childhood kidney cancer [84]. Tumor organoid biobanks have also been established for cervical [70] and lung [85] cancers, providing another reason to expect that research on these under-represented cancers will accelerate. In contrast to the above models with overall upward trends, genetically engineered mouse models (“GEMM”) have been less frequently mentioned in the past few years (Figure 4). In addition, overall reference to “murine” (including mice and rats) showed a slight downward trend (Figure 4; 10.0% relative decline across all tumor groups combined), which may reflect the expected advantage of human tumor organoid technology in reducing animal experimentation [86]. However, the widespread complementary use of xenograft models, as mentioned above, indicates mouse models are still a central part of tumor research.

An “assembloid” is a new type of composite organoid model made by assembling separate cell masses, often in the form of an organoid. However, to our knowledge, there are less than ten research articles using them as tumor models. For example, Choi et al. established pancreatic ductal adenocarcinoma assembloids composed of ductal cancer cells, endothelial cells, and autologous immune cells, and identified key players contributing to cancer cell plasticity and tumor heterogeneity [87]. Liang et al. used assembloids composed of cerebral cortical organoids and glioblastoma cells and showed that the cell adhesion molecule CD146 plays a role in the invasiveness of glioblastoma stem cells [88].

Among therapeutic approaches, “personalized medicine”, which includes models where treatment is customized for an individual patient or patient cohort, shows a moderate but above-average trend (Figure 4; 18% higher than the overall publication trend in the corpus) and is consistent with the increased use of patient-derived material. As a recent example, Mauri et al. established tumor organoids from a patient with oligometastatic colorectal cancer and used them for drug sensitivity testing in parallel with clinical care [89]. Subsequently, they took into consideration the results of the drug testing to determine the most effective therapeutic agents for the patient, which led to tumor shrinkage. Regarding therapeutic regimes tested on tumor organoids, “chemotherapy” is the most frequently mentioned therapy type, accompanied by the term “chemoresistance”, which poses a major challenge to virtually all tumor types (Figure 4). Although “chemotherapy” shows an overall above-average trend in its mentions (31% higher than the overall publication trend in the corpus), “immunotherapy” shows a stronger upward trend (59% higher). Immunotherapy aims to treat cancers by augmenting the patient’s own immune system, for example by (i) modifying and reintroducing autologous immune cells (“adoptive cell therapy”), (ii) “immune checkpoint” inhibitors to prevent immune escape of tumor cells, (iii) “monoclonal antibodies” specifically targeting tumor cells, or (iv) “oncolytic viruses” that preferentially kill tumors and release tumor-specific antigens that can trigger an immune response [90]. Tumor organoids are especially valuable in immunotherapy research because mouse models are not sufficiently predictive for immunotherapy due to major differences in the immune system [91], and responses to immunotherapy widely vary among patients which is something being addressed with patient-derived tumor organoids in personalized settings [92]. “Radiotherapy” and “neoadjuvant therapy” also show high upward trends (Figure 4; 100% and 65% higher, respectively). The upward trend of “neoadjuvant therapy” may be explained because this type of therapy is becoming a standard component of the pre-surgical management of many locally advanced tumors. However, as patients’ responses can be variable, neoadjuvant therapy requires good predictive models [8].

Among pharmacological terms, “drug development” is a common topic across a wide range of tumor organoid groups with an overall unchanging trend (Figure 4). Nevertheless, the term shows an upward trend for neural, mammary, and other tumors. “High-throughput” drug development/screening in particular shows an upward trend, reflecting efforts to develop more cost-efficient platforms for this purpose. “Drug testing” is another broadly mentioned pharmacological topic, and this is increasingly performed using patient-derived tumor organoids (28% of research articles on drug testing mentioned patient-derived tumor organoids between 2011 and 2019, whereas the percentage increased to 60% in 2020–present). “Drug delivery” is yet to be widely studied using tumor organoids (Figure 4), although drug research could benefit from understanding or improving drug penetration in tumor tissue.

Among research topics listed under “physiology”, the terms “tumorigenesis”, “cancer stem cell”, “tumor heterogeneity”, and “tumor microenvironment” are broadly mentioned across tumor groups (Figure 4). Cancer stem cells are cancer cells with stem cell-like characteristics, and have the ability to give rise to multiple cell types within a particular cancer [93]. As such, cancer stem cells can be an important driver of tumorigenesis, tumor progression, metastasis, and therapy resistance, and are hence considered to be an important therapeutic target. “Tumor heterogeneity” depicts genetic and phenotypic variations among tumors or within a tumor and is a major cause for therapy resistance and variations in the patients’ response to treatments. The advantage of tumor organoids compared to tumor cell lines is the ability to better recapitulate tumor heterogeneity, in particular when they are composed of cells from different areas of the tumor or its metastases [94]. The tumor microenvironment is the tissue architecture surrounding tumors. It is typically composed of stromal cells (including cancer-associated fibroblasts; CAF), immune cells, blood vessels (tumor endothelial cells), signaling molecules, and extracellular matrix [95]. The tumor microenvironment contributes to tumor survival and progression, metastasis, and therapy resistance by affecting immune reactions and supplying nutrients and oxygen through blood vessels. Reflecting its perceived importance, the tumor microenvironment is increasingly being studied using tumor organoids in a wide range of tumor groups (Figure 4), along with related physiological conditions and processes such as reduced oxygen levels (“hypoxia”), formation of blood vessels (“tumor angiogenesis”), “CAF”, and immune cells (see below). Tumor organoid technology can incorporate aspects of the tumor microenvironment, for example through air-liquid interface culture or co-culture with exogenous components such as immune cells and cancer-associated fibroblasts [96]. Although “angiogenesis” does not show an upward research trend, vascularization of tumor organoids in general has technically advanced in recent years, for example through the use of 3D bioprinting or ToC platforms [13].

“Metastasis” is the major cause of cancer-related deaths and occurs through; (i) an epithelial-mesenchymal transition (“EMT”) by which an epithelial (cancer) cell gains migratory and invasive properties, (ii) “intravasation” of the migratory cancer cell into the blood vessel, (iii) travel of the cancer cell in the blood as a “circulating tumor cell” to a new tissue location, (iv) “extravasation” whereby tumor cells translocate from the bloodstream to surrounding tissue, and (v) mesenchymal-epithelial transition (“MET”) by which the cancer cell regains epithelial properties to initiate metastatic cancer [23]. Tumor organoids have proven useful for studying these processes; for example they have been used to measure the degree of EMT and invasiveness to investigate the underlying molecular mechanisms [97]. Metastasis has been highly studied in context of breast and prostate cancers (Figure S4) with an upward trend for the latter (Figure 4), reflecting that these primary cancers are the top causes of metastasis in female and male, respectively [98]. The most common combinations of primary and metastatic cancers investigated using tumor organoid models are (i) large intestine—liver (41 research articles, out of 129 identified by the algorithm that were studying metastasis rather than merely mentioning the process), (ii) prostate—bone (14), (iii) mammary gland—lung (14), (iv) large intestine—lung (12), and (v) pancreas—liver (11). While in the clinic, the most common primary-metastatic interactions are: (i) prostate—bone, (ii) large intestine—liver, (iii) mammary gland—bone, (iv) mammary gland—liver, (v) mammary gland—lung, (vi) large intestine—lung, and (vii) lung—bone [98]. Thus, despite a relatively small number of research articles on metastasis, the researched combinations of primary and metastatic cancers somewhat mirror the clinical occurrence. The experimental approaches to study metastasis commonly include; (i) generation of matched tumor organoids from primary and metastatic cancers of the same patient (e.g., [73]), and (ii) generation of primary tumor organoids, combined with mouse models to study metastasis (e.g., [99]).

In addition, research topics related to host-microbe interactions have been gaining increasing attention (Figure 4). These topics include tumor microbiome [100], pro-oncogenic [101] and anti-oncogenic [102] effects of bacteria, as well as use of bacteria for cancer therapy [103].

A range of “immune cells” have been mentioned across the tumor organoid corpus, most of which show upward trends (Figure 4). These upward trends appear to reflect two emerging research foci, (i) incorporation of immune cells into tumor organoid models to better mimic the tumor microenvironment, and (ii) addressing immune cells in the context of immunotherapy. Terms grouped in “cellular processes” also show upward trends. “Apoptosis” is a major driver of cancer cell death [104]; “autophagy” contributes to both suppression and progression of tumor [105]; “ER stress” [106] and “reactive oxygen species” [107] are typically elevated in cancer cells, and therefore all these subcellular processes are (potential) targets of cancer therapy. “Extracellular vesicles” are studied to understand their roles in intercellular signaling, tumor progression, and chemoresistance, but also as a potential source of tumor biomarkers and possibly therapeutic agents [108]. Collectively, tumor physiology topics such as tumor heterogeneity and microenvironment remain important and are still a focus of research, while immune cells and (sub)cellular processes represent emerging research foci.

Among the listed techniques, “gene editing” is a key technique to introduce oncogenic mutations in (non-tumor) organoids to study tumorigenesis [109], although the publication trend is moderate (Figure 4). Upward publication trends are seen for a series of omics approaches, which reflects the wide recognition that tumor organoids sufficiently recapitulate the physiology of the original tumor for such analysis [110]. Especially, single-cell analysis such as single-cell RNA sequencing is increasingly performed (Figure 4) to study e.g. intra- and inter-patient tumor heterogeneity [111]. Other emerging analytical techniques include high-content analysis for high-throughput drug screening [112], Raman spectroscopy for biochemical phenotyping [113], while technical methods include 3D bioprinting to incorporate components of tumor microenvironments with spatial accuracy, as well as to develop high-throughput platforms [114].

### 3.6. Research Topics in ToC Research

**Figure 5:**
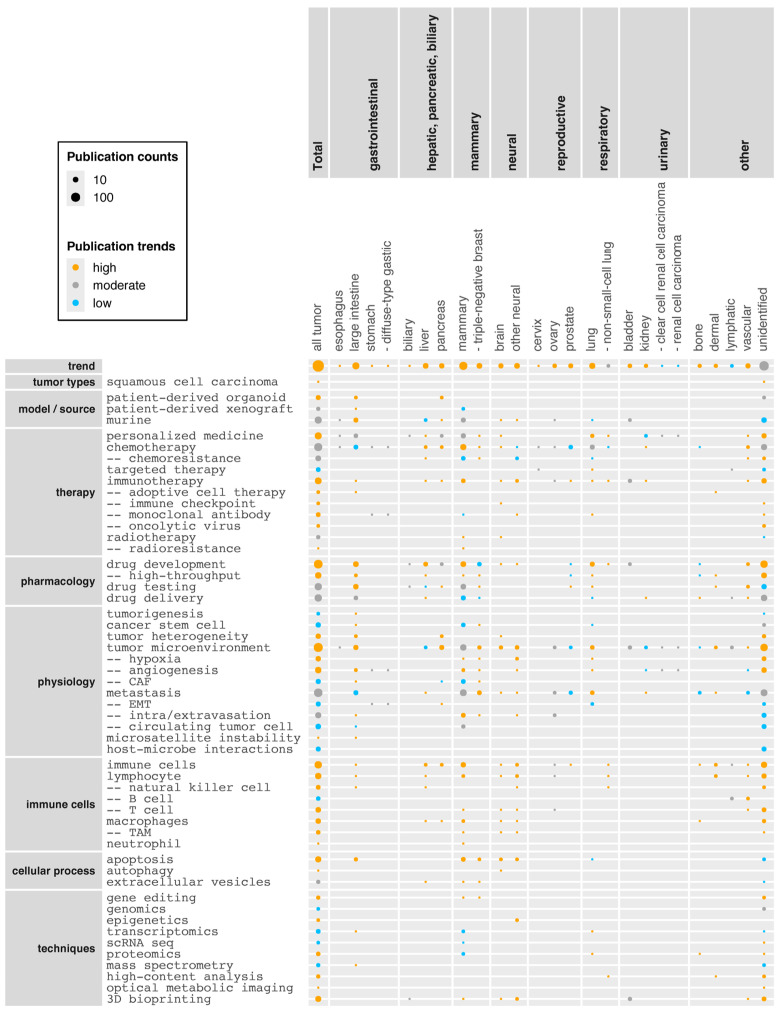
Trends of research topics in ToC research. The correlation matrix shows correlation between researched tumor groups (in *x*-axis) and selected research topics (in *y*-axis), along with their publication trends in ToC research.

Figure 5 highlights thematic topic trends in ToC research. Due to the small number of published articles compared to tumor organoids, trends may reflect stochastic changes rather than overall developments in the field. Nevertheless, trending topics are visible when all ToC tumor models are examined collectively (the leftmost column in Figure 5). Similar to tumor organoids, ToC platforms are used to model gastrointestinal, HPB, mammary, neural, reproductive, respiratory and urinary tumors. However, to date, only OoC but not ToC platforms have been developed as (neuro)endocrine, uterine, and oral models (Figure 5) [30]. Not surprisingly, tumor models that include blood vessel surrogates have been mostly developed as ToC (see “vascular” column). Although “personalized medicine” is increasingly mentioned in the context of ToC, the use of patient-derived material (e.g., “patient-derived organoids”) so far has been very limited for ToC platforms. Similar to tumor organoid research, “immunotherapy” represents the therapy type with a high upward trend that coincides with upward trends in delivering different types of immune cells. Indeed, ToC platforms are particularly suitable for tracking and studying tumor/immune interactions [115].

Consistent with the advantages of OoC platforms for pharmacological research [30,116], pharmacological topics are relatively more frequently mentioned in ToC research articles than tumor organoid articles (drug development: 13% in the tumor organoid corpus vs. 28% in the ToC corpus; high-throughput: 3.5% vs. 6.3%; drug delivery: 1.1% vs. 12%), although the frequency is comparable for drug testing (9.6% vs. 10%). Tumor microenvironment (10% vs. 34%) and metastasis (22% vs. 25%) are also frequently mentioned in the ToC corpus, reflecting the utility of OoC technology for these models [23,117]. For example, patient-derived pancreatic ductal adenocarcinoma organoids have been co-cultured in ToC platforms with either pancreatic stellate cells and macrophages to mimic aspects of the tumor microenvironment [118], or with primary human fibroblasts and endothelial cells for mimicking stroma components and vascularization [119]. Tumor organoid-on-chip models of metastasis have been developed, whereby colorectal cancer organoids, as well as cell lines representing liver, lung, and epithelial cells were cultured in separate chambers of a single microfluidic device, in which tumor cell migration was monitored [120]. Common combinations of primary and metastatic cancers that are investigated using ToC models include (i) mammary gland—liver (7 of 23 research articles studying metastasis using ToC), (ii) large intestine—liver (3), and (iii) mammary gland—bone (3), also reflecting the incidence of clinical occurrence (see above) [98].

Among the technical methods, 3D bioprinting shows an upward trend (Figure 5) and is more frequently used with ToC platforms rather than tumor organoids (4.9% vs. 0.8%). The technology is mainly used to study components of tumor microenvironments on ToC platforms [114], with a particular interest in incorporating tumor vasculature [121].

### 3.7. Global Distribution and Trends in Tumor Organoid and ToC Research

**Figure 6:**
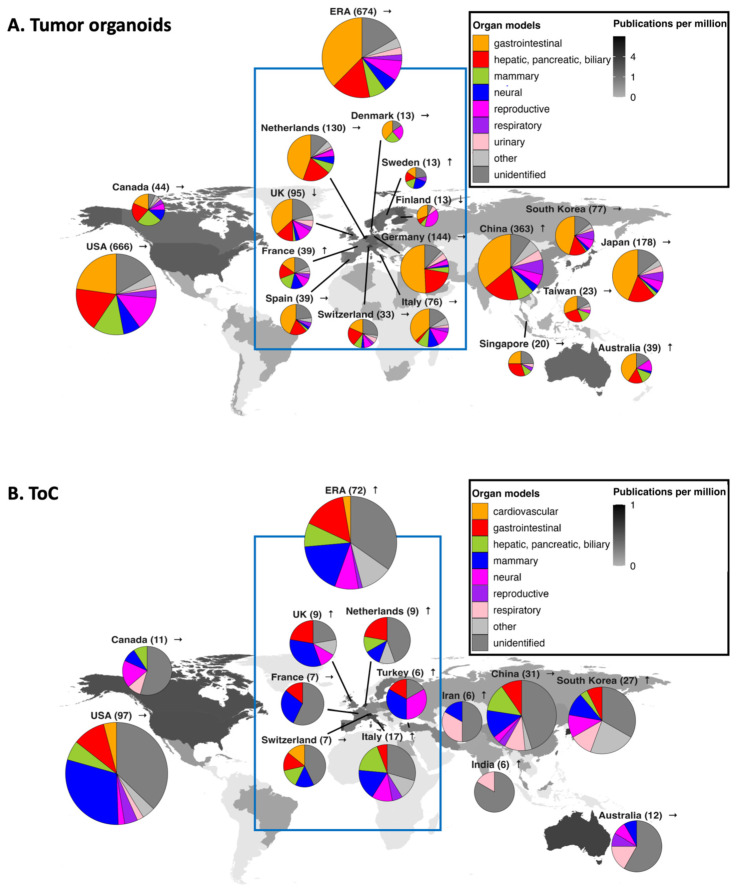
Global trends in tumor organoid and ToC research. World map and publication trends of countries/regions in (**A**) tumor organoid and (**B**) ToC research. Pie charts of top countries/regions are shown along with their number of research articles. Arrows indicate recent trends of publication counts. For better visibility of smaller pies, diameters of the pie charts are in a cube root scale such that the area nonlinearly correlates with the number of research articles. The greyscale gradient on the map shows the sum of fractional counts of contributions of each country, adjusted for the population size. “Unidentified” as an organ model category consists of research articles in which the algorithm did not detect an organ model type.

To analyze the geographical distribution of tumor organoid and ToC research, research articles were grouped under the first-level organ categories and attributed to countries/regions where the research was performed. The ERA and the USA are the top two contributors to tumor organoid research, accounting for 31.0% and 30.7% of research articles overall, respectively (Figure 6A). China is the third largest contributor, accounting for 16.7% of research articles, with a 3.8 fold higher increase than the global average in the recent publication count.

In the ERA, countries with the highest publication counts are Germany, the Netherlands, and the UK, while France and Sweden show recent above-average increases (Figure 6A). In Asia, Japan and South Korea follow China in total publication count. When research contributions are measured per capita (shown as a greyscale of the country map), the Netherlands, followed by Switzerland and Singapore show the highest publication activity. The Netherlands, a pioneer in organoid technology, also shows strong research activity in tumor organoid research, especially for colorectal cancer. Collectively, the global distribution of tumor organoid research closely mirrors that of non-tumor organoid research [30].

A majority of countries/regions share comparable distributions of published organ models in tumor organoid research, with gastrointestinal tumor organoids being by far the most studied, followed by HPB models (Figure 6A). Respiratory tumor organoids are comparatively well-researched in Asian countries, possibly due to the high incidence/mortality of lung cancer in these regions [2]. Conversely, even though breast cancer is especially prevalent in Australia/New Zealand, Europe, and North America [2], mammary tumor organoid research is no more active in these regions compared to other regions, such as China and Taiwan in East Asia. Likewise, prostate cancer is common in northern and western Europe, as well as in North America, but is less common in eastern Asia [2]. However, to date, prostate tumor organoid research has predominantly been performed in the USA (63 publications), followed by China (8) and the UK (6), with lower research output from other European countries. Overall, we did not note a clear presence of a national focus on tumor organoid research that reflects regional cancer burden.

In ToC research, a similar overall pattern is seen for the most productive countries/regions, with a few exceptions (Figure 6B). The top country is the USA (accounting for 34% of research articles), followed by the ERA (25%, of which Italy contributes 6%), China (11%) and South Korea (9%). Countries/regions with upward trends in the publication counts include the ERA, Italy, and South Korea, which is noteworthy considering their publication trends in tumor organoid research are flat. When research contributions are measured per capita, Switzerland, followed by the Netherlands and South Korea, shows the highest activity in ToC research.

## 4. Conclusions

In this study, we analyzed approximately 3500 academic publications on tumor organoids and ToC, complementing our previous analysis of general organoid and OoC literature. Our text-analysis approach does have weaknesses, including its reliance on data sources such as article type classifications at publishers and the terminology used by publication authors [30]. For example, our ToC corpus lacks publications which do not use the terms “on-chip” or “microphysiological system” to describe culturing tumor cells on microfluidic devices. This limitation is because database searches using terms “tumor” and “microfluidics” or similar resulted in many publications that do not culture tumor cells but are still related to tumor research. In this regard, our analysis is largely limited to research areas and subareas where terminology is well-defined, which potentially could lead to analytical bias. In addition, our analysis is prone to misclassification and misinterpretation due to general limitations of natural language processing approaches. Nevertheless, our method allows identification of research activities and trends in a quantitative and reproducible manner, thereby highlighting emerging and under-studied research areas. Such knowledge can be used by researchers as a rational basis for future research and grant applications.

Tumor organoid research is currently in an expansion stage, with established research subareas such as the large intestine, HPB, and prostate tumor organoids which are well-represented in comparison to their global incidence and mortality rates. Research is also expanding with newly emerging tumor organoid models such as thyroid, musculoskeletal, oral, and ovarian tumor organoids for studying less common tumors. Additionally, there is a growing focus on models of under-represented tumors such as lung and cervical cancers. Therapeutic approaches, particularly immunotherapy and within personalized medicine, are also expanding to include innovative methods; for example, to incorporate immune cells into some models. Finally, research is also delving deeper into studying tumor physiology at (sub)cellular levels.

Although the ToC corpus was smaller, it still indicates robust growth in publication volume. ToC platforms are proving particularly effective for incorporating components of the tumor microenvironment, studying metastasis, and examining tumor-immune cell interactions [23,117]. We anticipate these areas to see increased research activity going forward.

Clinical applications of tumor organoids still face many challenges, including variable reproducibility, relatively high costs, limitations in recapitulating the tumor microenvironment including difficulties in modeling vasculature and immune interface [122]. Nevertheless, our analysis shows increasing research activities to address some of these challenges. Furthermore, ToC models appear to be increasingly studied to counter these limitations of tumor organoids, for example as a cost-efficient platform for high-throughput drug screening, or by incorporating tumor microenvironment components or vascularization/endothelialization-enabling microfluidic channels. In addition, by also considering recent research developments in organoid and OoC technologies in general [30], we expect tumor organoid and ToC research to advance in several key areas: (i) increased use of ToC platforms to more accurately simulate the tumor microenvironment and study complex interactions including metastasis and immune responses; (ii) development of personalized tumor models in underrepresented tumor areas; and (iii) expansion of tumor organoid and ToC as high-throughput platforms for drug development and testing.

In summary, our analysis suggests tumor organoids are becoming indispensable model systems and are poised to become a routine tool in the near future. ToC models are also rapidly evolving and are likely to closely follow and complement tumor organoid models in their integral role in advancing our understanding of cancer.

## Data Availability

The code used for the analysis is available at the GitHub repository (https://github.com/jyshoji/text_analysis_tumor_organoids (accessed on 20 December 2024); DOI: 10.5281/zenodo.13890271). The underlying data were obtained from EMBASE (https://ovidsp.ovid.com), PubMed (https://pubmed.ncbi.nlm.nih.gov), Scopus (https://www.scopus.com/), Web of Science (https://www.webofscience.com/), and bioRxiv (https://www.biorxiv.org), and are available from the URLs with the permission of these databases. Additionally, we provide in the repository a subset of data that we can publicly share, including: (i) a list of DOIs and titles of the academic publications along with full results of the analysis, and (ii) a subset of publication metadata under the Creative Commons Attribution Licenses which can be readily analyzed by the code. The complete corpus we used for the analysis is available upon request to the lead contact Stefan Krauss (s.j.k.krauss@medisin.uio.no).

## Supplementary Materials

The following supporting information can be downloaded at: https://www.mdpi.com/article/10.3390/cancers17010108/s1, Figure S1: The number of clinical trials using tumor organoids and/or ToC; Figure S2: Hierarchical arrangement of organ and organ substructures modelled as tumor organoids; Figure S3: Hierarchical arrangement of organ and organ substructures modelled as ToC; Figure S4: Research topic occurrences by tumor types in tumor organoid research; Table S1: Publications on tumor organoid models and their organ/substructure classifications; Table S2: Publications on ToC models and their organ/substructure classifications; Table S3: Publications on tumor organoid and ToC models and their research topics.

## References

[1] Bray F., Laversanne M., Weiderpass E., Soerjomataram I. (2021). The ever-increasing importance of cancer as a leading cause of premature death worldwide. Cancer.

[2] Sung H., Ferlay J., Siegel R.L., Laversanne M., Soerjomataram I., Jemal A., Bray F. (2021). Global Cancer Statistics 2020: GLOBOCAN Estimates of Incidence and Mortality Worldwide for 36 Cancers in 185 Countries. CA Cancer J. Clin..

[3] Bleijs M., van de Wetering M., Clevers H., Drost J. (2019). Xenograft and organoid model systems in cancer research. EMBO J..

[4] Wlodkowic D., Cooper J.M. (2010). Tumors on chips: Oncology meets microfluidics. Curr. Opin. Chem. Biol..

[5] Sachs N., Clevers H. (2014). Organoid cultures for the analysis of cancer phenotypes. Curr. Opin. Genet. Dev..

[6] Frese K.K., Tuveson D.A. (2007). Maximizing mouse cancer models. Nat. Rev. Cancer.

[7] Tuveson D., Clevers H. (2019). Cancer modeling meets human organoid technology. Science.

[8] Gunti S., Hoke A.T.K., Vu K.P., London N.R.J. (2021). Organoid and Spheroid Tumor Models: Techniques and Applications. Cancers.

[9] Marsee A., Roos F.J.M., Verstegen M.M.A., Gehart H., de Koning E., Lemaigre F., Forbes S.J., Peng W.C., Huch M., Takebe T. (2021). Building consensus on definition and nomenclature of hepatic, pancreatic, and biliary organoids. Cell Stem Cell.

[10] Eiraku M., Watanabe K., Matsuo-Takasaki M., Kawada M., Yonemura S., Matsumura M., Wataya T., Nishiyama A., Muguruma K., Sasai Y. (2008). Self-organized formation of polarized cortical tissues from ESCs and its active manipulation by extrinsic signals. Cell Stem Cell.

[11] Sato T., Vries R.G., Snippert H.J., van de Wetering M., Barker N., Stange D.E., van Es J.H., Abo A., Kujala P., Peters P.J. (2009). Single Lgr5 stem cells build crypt-villus structures in vitro without a mesenchymal niche. Nature.

[12] Sato T., Stange D.E., Ferrante M., Vries R.G.J., Van Es J.H., Van den Brink S., Van Houdt W.J., Pronk A., Van Gorp J., Siersema P.D. (2011). Long-term expansion of epithelial organoids from human colon, adenoma, adenocarcinoma, and Barrett’s epithelium. Gastroenterology.

[13] Luo Z., Zhou X., Mandal K., He N., Wennerberg W., Qu M., Jiang X., Sun W., Khademhosseini A. (2021). Reconstructing the tumor architecture into organoids. Adv. Drug Deliv. Rev..

[14] Sachs N., de Ligt J., Kopper O., Gogola E., Bounova G., Weeber F., Balgobind A.V., Wind K., Gracanin A., Begthel H. (2018). A Living Biobank of Breast Cancer Organoids Captures Disease Heterogeneity. Cell.

[15] Boj S.F., Hwang C.-I., Baker L.A., Chio I.I.C., Engle D.D., Corbo V., Jager M., Ponz-Sarvise M., Tiriac H., Spector M.S. (2015). Organoid models of human and mouse ductal pancreatic cancer. Cell.

[16] Drost J., Karthaus W.R., Gao D., Driehuis E., Sawyers C.L., Chen Y., Clevers H. (2016). Organoid culture systems for prostate epithelial and cancer tissue. Nat. Protoc..

[17] Broutier L., Mastrogiovanni G., Verstegen M.M., Francies H.E., Gavarró L.M., Bradshaw C.R., Allen G.E., Arnes-Benito R., Sidorova O., Gaspersz M.P. (2017). Human primary liver cancer-derived organoid cultures for disease modeling and drug screening. Nat. Med..

[18] Hubert C.G., Rivera M., Spangler L.C., Wu Q., Mack S.C., Prager B.C., Couce M., McLendon R.E., Sloan A.E., Rich J.N. (2016). A Three-Dimensional Organoid Culture System Derived from Human Glioblastomas Recapitulates the Hypoxic Gradients and Cancer Stem Cell Heterogeneity of Tumors Found In Vivo. Cancer Res..

[19] Jung D.J., Shin T.H., Kim M., Sung C.O., Jang S.J., Jeong G.S. (2019). A one-stop microfluidic-based lung cancer organoid culture platform for testing drug sensitivity. Lab. Chip.

[20] Yan H.H.N., Siu H.C., Law S., Ho S.L., Yue S.S.K., Tsui W.Y., Chan D., Chan A.S., Ma S., Lam K.O. (2018). A Comprehensive Human Gastric Cancer Organoid Biobank Captures Tumor Subtype Heterogeneity and Enables Therapeutic Screening. Cell Stem Cell.

[21] Kopper O., de Witte C.J., Lõhmussaar K., Valle-Inclan J.E., Hami N., Kester L., Balgobind A.V., Korving J., Proost N., Begthel H. (2019). An organoid platform for ovarian cancer captures intra- and interpatient heterogeneity. Nat. Med..

[22] Lee S.H., Hu W., Matulay J.T., Silva M.V., Owczarek T.B., Kim K., Chua C.W., Barlow L.J., Kandoth C., Williams A.B. (2018). Tumor Evolution and Drug Response in Patient-Derived Organoid Models of Bladder Cancer. Cell.

[23] Del Piccolo N., Shirure V.S., Bi Y., Goedegebuure S.P., Gholami S., Hughes C.C.W., Fields R.C., George S.C. (2021). Tumor-on-chip modeling of organ-specific cancer and metastasis. Adv. Drug Deliv. Rev..

[24] Ying L., Zhu Z., Xu Z., He T., Li E., Guo Z., Liu F., Jiang C., Wang Q. (2015). Cancer Associated Fibroblast-Derived Hepatocyte Growth Factor Inhibits the Paclitaxel-Induced Apoptosis of Lung Cancer A549 Cells by Up-Regulating the PI3K/Akt and GRP78 Signaling on a Microfluidic Platform. PLoS ONE.

[25] Choi Y., Hyun E., Seo J., Blundell C., Kim H.C., Lee E., Lee S.H., Moon A., Moon W.K., Huh D. (2015). A microengineered pathophysiological model of early-stage breast cancer. Lab. Chip.

[26] Kerr S.C., Morgan M.M., Gillette A.A., Livingston M.K., Lugo-Cintron K.M., Favreau P.F., Florek L., Johnson B.P., Lang J.M., Skala M.C. (2020). A bioengineered organotypic prostate model for the study of tumor microenvironment-induced immune cell activation. Integr. Biol..

[27] Carvalho M.R., Barata D., Teixeira L.M., Giselbrecht S., Reis R.L., Oliveira J.M., Truckenmüller R., Habibovic P. (2019). Colorectal tumor-on-a-chip system: A 3D tool for precision onco-nanomedicine. Sci. Adv..

[28] Lee J.-H., Kim S.-K., Khawar I.A., Jeong S.-Y., Chung S., Kuh H.-J. (2018). Microfluidic co-culture of pancreatic tumor spheroids with stellate cells as a novel 3D model for investigation of stroma-mediated cell motility and drug resistance. J. Exp. Clin. Cancer Res..

[29] Abaci H.E., Gledhill K., Guo Z., Christiano A.M., Shuler M.L. (2015). Pumpless microfluidic platform for drug testing on human skin equivalents. Lab. Chip.

[30] Shoji J.-Y., Davis R.P., Mummery C.L., Krauss S. (2024). Global Literature Analysis of Organoid and Organ-on-Chip Research. Adv. Heal. Mater..

[31] R Core Team (2021). R: A Language and Environment for Statistical Computing.

[32] RStudio Team (2020). RStudio: Integrated Development Environment for R.

[33] Wickham H., Averick M., Bryan J., Chang W., McGowan L.D., François R., Grolemund G., Hayes A., Henry L., Hester J. (2019). Welcome to the tidyverse. J. Open Source Softw..

[34] Westgate M.J. (2019). revtools: An R package to support article screening for evidence synthesis. Res. Synth. Methods.

[35] Wikipedia (2022). List of Countries and Dependencies by Population. https://en.wikipedia.org/wiki/List_of_countries_and_dependencies_by_population.

[36] Csardi G., Nepusz T. (2006). The igraph software package for complex network research. InterJ. Complex Syst..

[37] Aphalo P.J. (2022). ggpp: Grammar Extensions to “ggplot2”. https://CRAN.R-project.org/package=ggpp.

[38] Pedersen T.L. (2021). ggraph: An Implementation of Grammar of Graphics for Graphs and Networks. https://CRAN.R-project.org/package=ggraph.

[39] South A. (2011). rworldmap: A New R package for Mapping Global Data. R. J..

[40] Holtz Y. The R Graph Gallery. https://r-graph-gallery.com.

[41] Nguyen A., McAleavey K., Lyall K. (2019). SnapShot: Advancing Organoid Technology. Cell Stem Cell.

[42] Chen Q., Suzuki K., Sifuentes-Dominguez L., Miyata N., Song J., Lopez A., Starokadomskyy P., Gopal P., Dozmorov I., Tan S. (2021). Paneth cell-derived growth factors support tumorigenesis in the small intestine. Life Sci. Alliance.

[43] Alexander K.L., Serrano C.A., Chakraborty A., Nearing M., Council L.N., Riquelme A., Garrido M., Bellis S.L., Smythies L.E., Smith P.D. (2020). Modulation of glycosyltransferase ST6Gal-I in gastric cancer-derived organoids disrupts homeostatic epithelial cell turnover. J. Biol. Chem..

[44] Wang H., Calvisi D.F., Chen X. (2021). Organoids for the Study of Liver Cancer. Semin. Liver Dis..

[45] García P., Rosa L., Vargas S., Weber H., Espinoza J.A., Suárez F., Romero-Calvo I., Elgueta N., Rivera V., Nervi B. (2020). Hippo-YAP1 Is a Prognosis Marker and Potentially Targetable Pathway in Advanced Gallbladder Cancer. Cancers.

[46] April-Monn S.L., Wiedmer T., Skowronska M., Maire R., Schiavo Lena M., Trippel M., Di Domenico A., Muffatti F., Andreasi V., Capurso G. (2021). Three-Dimensional Primary Cell Culture: A Novel Preclinical Model for Pancreatic Neuroendocrine Tumors. Neuroendocrinology.

[47] Rybin M.J., Ivan M.E., Ayad N.G., Zeier Z. (2021). Organoid Models of Glioblastoma and Their Role in Drug Discovery. Front. Cell Neurosci..

[48] Zhang D., Hugo W., Redublo P., Miao H., Bergsneider M., Wang M.B., Kim W., Yong W.H., Heaney A.P. (2021). A human ACTH-secreting corticotroph tumoroid model: Novel Human ACTH-Secreting Tumor Cell in vitro Model. EBioMedicine.

[49] Vincent-Chong V.K., Seshadri M. (2020). Development and Radiation Response Assessment in A Novel Syngeneic Mouse Model of Tongue Cancer: 2D Culture, 3D Organoids and Orthotopic Allografts. Cancers.

[50] Sondorp L.H.J., Ogundipe V.M.L., Groen A.H., Kelder W., Kemper A., Links T.P., Coppes R.P., Kruijff S. (2020). Patient-Derived Papillary Thyroid Cancer Organoids for Radioactive Iodine Refractory Screening. Cancers.

[51] Wei H., Li Y., Li L., Hu Q., Shi M., Cheng L., Jiang X., Zhou Y., Chen S., Ji Y. (2023). Novel organoid construction strategy for non-involuting congenital hemangioma for drug validation. J. Biol. Eng..

[52] Boulay G., Cironi L., Garcia S.P., Rengarajan S., Xing Y.-H., Lee L., Awad M.E., Naigles B., Iyer S., Broye L.C. (2021). The chromatin landscape of primary synovial sarcoma organoids is linked to specific epigenetic mechanisms and dependencies. Life Sci. Alliance.

[53] Takada K., Aizawa Y., Sano D., Okuda R., Sekine K., Ueno Y., Yamanaka S., Aoyama J., Sato K., Kuwahara T. (2021). Establishment of PDX-derived salivary adenoid cystic carcinoma cell lines using organoid culture method. Int. J. Cancer.

[54] Lucky S.S., Law M., Lui M.H., Mong J., Shi J., Yu S., Yoon D.K., Djeng S.K., Wang J., Lim C.M. (2021). Patient-Derived Nasopharyngeal Cancer Organoids for Disease Modeling and Radiation Dose Optimization. Front. Oncol..

[55] Li S., Lee D.-J., Kim H.-Y., Kim J.-Y., Jung Y.-S., Jung H.-S. (2022). Ameloblastoma modifies tumor microenvironment for enhancing invasiveness by altering collagen alignment. Histochem. Cell Biol..

[56] Saha S., Howarth R., Sharma-Saha S., Kelly C. (2023). Bioengineering of a tumour-stroma 3D-tumouroid co-culture model of hypopharyngeal cancer. Biol. Open.

[57] Pavlou M., Shah M., Gikas P., Briggs T., Roberts S.J., Cheema U. (2019). Osteomimetic matrix components alter cell migration and drug response in a 3D tumour-engineered osteosarcoma model. Acta Biomater..

[58] Veys C., Benmoussa A., Contentin R., Duchemin A., Brotin E., Lafont J.E., Saintigny Y., Poulain L., Denoyelle C., Demoor M. (2021). Tumor Suppressive Role of miR-342-5p in Human Chondrosarcoma Cells and 3D Organoids. Int. J. Mol. Sci..

[59] Hu Z., Cao Y., Galan E.A., Hao L., Zhao H., Tang J., Sang G., Wang H., Xu B., Ma S. (2022). Vascularized Tumor Spheroid-on-a-Chip Model Verifies Synergistic Vasoprotective and Chemotherapeutic Effects. ACS Biomater. Sci. Eng..

[60] Lee S.-Y., Byeon S., Ko J., Hyung S., Lee I.-K., Jeon N.L., Hong J.Y., Kim S.T., Park S.H., Lee J. (2021). Reducing tumor invasiveness by ramucirumab and TGF-β receptor kinase inhibitor in a diffuse-type gastric cancer patient-derived cell model. Cancer Med..

[61] Liu Q., Mille L.S., Villalobos C., Anaya I., Vostatek M., Yi S., Li W., Liao J., Wu H., Song Y. (2023). 3D-bioprinted cholangiocarcinoma-on-a-chip model for evaluating drug responses. Bio-Des. Manuf..

[62] Lin C., Lin L., Mao S., Yang L., Yi L., Lin X., Wang J., Lin Z.-X., Lin J.-M. (2018). Reconstituting Glioma Perivascular Niches on a Chip for Insights into Chemoresistance of Glioma. Anal. Chem..

[63] Yi H.-G., Jeong Y.H., Kim Y., Choi Y.-J., Moon H.E., Park S.H., Kang K.S., Bae M., Jang J., Youn H. (2019). A bioprinted human-glioblastoma-on-a-chip for the identification of patient-specific responses to chemoradiotherapy. Nat. Biomed. Eng..

[64] Marzagalli M., Pelizzoni G., Fedi A., Vitale C., Fontana F., Bruno S., Poggi A., Dondero A., Aiello M., Castriconi R. (2022). A multi-organ-on-chip to recapitulate the infiltration and the cytotoxic activity of circulating NK cells in 3D matrix-based tumor model. Front. Bioeng. Biotechnol..

[65] Ma C., Witkowski M.T., Harris J., Dolgalev I., Sreeram S., Qian W., Tong J., Chen X., Aifantis I., Chen W. (2020). Leukemia-on-a-chip: Dissecting the chemoresistance mechanisms in B cell acute lymphoblastic leukemia bone marrow niche. Sci. Adv..

[66] Mitxelena-Iribarren O., Lizarbe-Sancha S., Campisi J., Arana S., Mujika M. (2021). Different Microfluidic Environments for In Vitro Testing of Lipid Nanoparticles against Osteosarcoma. Bioengineering.

[67] Saha B., Mathur T., Handley K.F., Hu W., Afshar-Kharghan V., Sood A.K., Jain A. (2020). OvCa-Chip microsystem recreates vascular endothelium-mediated platelet extravasation in ovarian cancer. Blood Adv..

[68] Kim J.H., Lee S., Kang S.J., Choi Y.W., Choi S.Y., Park J.Y., Chang I.H. (2021). Establishment of Three-Dimensional Bioprinted Bladder Cancer-on-a-Chip with a Microfluidic System Using Bacillus Calmette-Guérin. Int. J. Mol. Sci..

[69] American Cancer Society Survival Rates for Pancreatic Cancer. https://www.cancer.org/cancer/types/pancreatic-cancer/detection-diagnosis-staging/survival-rates.html.

[70] Lõhmussaar K., Oka R., Espejo Valle-Inclan J., Smits M.H.H., Wardak H., Korving J., Begthel H., Proost N., van de Ven M., Kranenburg O.W. (2021). Patient-derived organoids model cervical tissue dynamics and viral oncogenesis in cervical cancer. Cell Stem Cell.

[71] Li Y., Wang R., Huang D., Ma X., Mo S., Guo Q., Fu G., Li Y., Xu X., Hu X. (2019). A novel human colon signet-ring cell carcinoma organoid line: Establishment, characterization and application. Carcinogenesis.

[72] Verduin M., Hoeben A., De Ruysscher D., Vooijs M. (2021). Patient-Derived Cancer Organoids as Predictors of Treatment Response. Front. Oncol..

[73] Mo S., Tang P., Luo W., Zhang L., Li Y., Hu X., Ma X., Chen Y., Bao Y., He X. (2022). Patient-Derived Organoids from Colorectal Cancer with Paired Liver Metastasis Reveal Tumor Heterogeneity and Predict Response to Chemotherapy. Adv. Sci..

[74] Maier C.F., Zhu L., Nanduri L.K., Kühn D., Kochall S., Thepkaysone M.-L., William D., Grützmann K., Klink B., Betge J. (2021). Patient-Derived Organoids of Cholangiocarcinoma. Int. J. Mol. Sci..

[75] Gao D., Vela I., Sboner A., Iaquinta P.J., Karthaus W.R., Gopalan A., Dowling C., Wanjala J.N., Undvall E.A., Arora V.K. (2014). Organoid cultures derived from patients with advanced prostate cancer. Cell.

[76] Lawrence M.G., Taylor R.A., Cuffe G.B., Ang L.S., Clark A.K., Goode D.L., Porter L.H., Le Magnen C., Navone N.M., Schalken J.A. (2023). The future of patient-derived xenografts in prostate cancer research. Nat. Rev. Urol..

[77] De Angelis M.L., Francescangeli F., Nicolazzo C., Signore M., Giuliani A., Colace L., Boe A., Magri V., Baiocchi M., Ciardi A. (2022). An organoid model of colorectal circulating tumor cells with stem cell features, hybrid EMT state and distinctive therapy response profile. J. Exp. Clin. Cancer Res..

[78] Wakamatsu T., Ogawa H., Yoshida K., Matsuoka Y., Shizuma K., Imura Y., Tamiya H., Nakai S., Yagi T., Nagata S. (2022). Establishment of Organoids From Human Epithelioid Sarcoma With the Air-Liquid Interface Organoid Cultures. Front. Oncol..

[79] Li H., Liu H., Chen K. (2022). Living biobank-based cancer organoids: Prospects and challenges in cancer research. Cancer Biol. Med..

[80] Kawasaki K., Toshimitsu K., Matano M., Fujita M., Fujii M., Togasaki K., Ebisudani T., Shimokawa M., Takano A., Takahashi S. (2020). An Organoid Biobank of Neuroendocrine Neoplasms Enables Genotype-Phenotype Mapping. Cell.

[81] Huang B., Trujillo M.A., Fujikura K., Qiu M., Chen F., Felsenstein M., Zhou C., Skaro M., Gauthier C., Macgregor-Das A. (2020). Molecular characterization of organoids derived from pancreatic intraductal papillary mucinous neoplasms. J. Pathol..

[82] Jacob F., Salinas R.D., Zhang D.Y., Nguyen P.T.T., Schnoll J.G., Wong S.Z.H., Thokala R., Sheikh S., Saxena D., Prokop S. (2020). A Patient-Derived Glioblastoma Organoid Model and Biobank Recapitulates Inter- and Intra-tumoral Heterogeneity. Cell.

[83] Wang X.-W., Xia T.-L., Tang H.-C., Liu X., Han R., Zou X., Zhao Y.-T., Chen M.-Y., Li G. (2022). Establishment of a patient-derived organoid model and living biobank for nasopharyngeal carcinoma. Ann. Transl. Med..

[84] Calandrini C., Schutgens F., Oka R., Margaritis T., Candelli T., Mathijsen L., Ammerlaan C., van Ineveld R.L., Derakhshan S., de Haan S. (2020). An organoid biobank for childhood kidney cancers that captures disease and tissue heterogeneity. Nat. Commun..

[85] Li Y.F., Gao Y., Liang B.W., Cao X.Q., Sun Z.J., Yu J.H., Liu Z.D., Han Y. (2020). Patient-derived organoids of non-small cells lung cancer and their application for drug screening. Neoplasma.

[86] Tosca E.M., Ronchi D., Facciolo D., Magni P. (2023). Replacement, Reduction, and Refinement of Animal Experiments in Anticancer Drug Development: The Contribution of 3D In Vitro Cancer Models in the Drug Efficacy Assessment. Biomedicines.

[87] Choi J.-I., Rim J.H., Jang S.I., Park J.S., Park H., Cho J.H., Lim J.-B. (2022). The role of Jagged1 as a dynamic switch of cancer cell plasticity in PDAC assembloids. Theranostics.

[88] Liang Y., Voshart D., Paridaen J.T.M.L., Oosterhof N., Liang D., Thiruvalluvan A., Zuhorn I.S., den Dunnen W.F.A., Zhang G., Lin H. (2022). CD146 increases stemness and aggressiveness in glioblastoma and activates YAP signaling. Cell Mol. Life Sci..

[89] Mauri G., Durinikova E., Amatu A., Tosi F., Cassingena A., Rizzetto F., Buzo K., Arcella P., Aquilano M.C., Bonoldi E. (2021). Empowering Clinical Decision Making in Oligometastatic Colorectal Cancer: The Potential Role of Drug Screening of Patient-Derived Organoids. JCO Precis. Oncol..

[90] Zhang Y., Zhang Z. (2020). The history and advances in cancer immunotherapy: Understanding the characteristics of tumor-infiltrating immune cells and their therapeutic implications. Cell. Mol. Immunol..

[91] Grönholm M., Feodoroff M., Antignani G., Martins B., Hamdan F., Cerullo V. (2021). Patient-Derived Organoids for Precision Cancer Immunotherapy. Cancer Res..

[92] Boucherit N., Gorvel L., Olive D. (2020). 3D Tumor Models and Their Use for the Testing of Immunotherapies. Front. Immunol..

[93] Batlle E., Clevers H. (2017). Cancer stem cells revisited. Nat. Med..

[94] Roerink S.F., Sasaki N., Lee-Six H., Young M.D., Alexandrov L.B., Behjati S., Mitchell T.J., Grossmann S., Lightfoot H., Egan D.A. (2018). Intra-tumour diversification in colorectal cancer at the single-cell level. Nature.

[95] Chen F., Zhuang X., Lin L., Yu P., Wang Y., Shi Y., Hu G., Sun Y. (2015). New horizons in tumor microenvironment biology: Challenges and opportunities. BMC Med..

[96] Xia T., Du W.-L., Chen X.-Y., Zhang Y.-N. (2021). Organoid models of the tumor microenvironment and their applications. J. Cell Mol. Med..

[97] Jung H.-Y., Fattet L., Tsai J.H., Kajimoto T., Chang Q., Newton A.C., Yang J. (2019). Apical-basal polarity inhibits epithelial-mesenchymal transition and tumour metastasis by PAR-complex-mediated SNAI1 degradation. Nat. Cell Biol..

[98] Riihimäki M., Thomsen H., Sundquist K., Sundquist J., Hemminki K. (2018). Clinical landscape of cancer metastases. Cancer Med..

[99] Li X., Li J., Xu L., Wei W., Cheng A., Zhang L., Zhang M., Wu G., Cai C. (2022). CDK16 promotes the progression and metastasis of triple-negative breast cancer by phosphorylating PRC1. J. Exp. Clin. Cancer Res..

[100] Chen Y., Yang S., Tavormina J., Tampe D., Zeisberg M., Wang H., Mahadevan K.K., Wu C.-J., Sugimoto H., Chang C.-C. (2022). Oncogenic collagen I homotrimers from cancer cells bind to α3β1 integrin and impact tumor microbiome and immunity to promote pancreatic cancer. Cancer Cell.

[101] Cao L., Zhu S., Lu H., Soutto M., Bhat N., Chen Z., Peng D., Lin J., Lu J., Li P. (2022). Helicobacter pylori-induced RASAL2 Through Activation of Nuclear Factor-κB Promotes Gastric Tumorigenesis via β-catenin Signaling Axis. Gastroenterology.

[102] Sugimura N., Li Q., Chu E.S.H., Lau H.C.H., Fong W., Liu W., Liang C., Nakatsu G., Su A.C.Y., Coker O.O. (2021). Lactobacillus gallinarum modulates the gut microbiota and produces anti-cancer metabolites to protect against colorectal tumourigenesis. Gut.

[103] Mackie G.M., Copland A., Takahashi M., Nakanishi Y., Everard I., Kato T., Oda H., Kanaya T., Ohno H., Maslowski K.M. (2021). Bacterial cancer therapy in autochthonous colorectal cancer affects tumor growth and metabolic landscape. JCI Insight.

[104] Pfeffer C.M., Singh A.T.K. (2018). Apoptosis: A Target for Anticancer Therapy. Int. J. Mol. Sci..

[105] Yun C.W., Lee S.H. (2018). The Roles of Autophagy in Cancer. Int. J. Mol. Sci..

[106] Chen X., Cubillos-Ruiz J.R. (2021). Endoplasmic reticulum stress signals in the tumour and its microenvironment. Nat. Rev. Cancer.

[107] Nakamura H., Takada K. (2021). Reactive oxygen species in cancer: Current findings and future directions. Cancer Sci..

[108] Bordanaba-Florit G., Madarieta I., Olalde B., Falcón-Pérez J.M., Royo F. (2021). 3D Cell Cultures as Prospective Models to Study Extracellular Vesicles in Cancer. Cancers.

[109] Chen C., Ji W., Niu Y. (2021). Primate Organoids and Gene-Editing Technologies toward Next-Generation Biomedical Research. Trends Biotechnol..

[110] Ha D., Kong J., Kim D., Lee K., Lee J., Park M., Ahn H., Oh Y., Kim S. (2023). Development of bioinformatics and multi-omics analyses in organoids. BMB Rep..

[111] Landon-Brace N., Li N.T., McGuigan A.P. (2023). Exploring New Dimensions of Tumor Heterogeneity: The Application of Single Cell Analysis to Organoid-Based 3D In Vitro Models. Adv. Heal. Mater..

[112] Wang Y., Liao H., Zheng T., Wang J., Guo D., Lu Z., Li Z., Chen Y., Shen L., Zhang Y. (2020). Conditionally reprogrammed colorectal cancer cells combined with mouse avatars identify synergy between EGFR and MEK or CDK4/6 inhibitors. Am. J. Cancer Res..

[113] Jamieson L.E., Harrison D.J., Campbell C.J. (2019). Raman spectroscopy investigation of biochemical changes in tumor spheroids with aging and after treatment with staurosporine. J. Biophotonics.

[114] Shukla P., Yeleswarapu S., Heinrich M.A., Prakash J., Pati F. (2022). Mimicking tumor microenvironment by 3D bioprinting: 3D cancer modeling. Biofabrication.

[115] Parlato S., Grisanti G., Sinibaldi G., Peruzzi G., Casciola C.M., Gabriele L. (2021). Tumor-on-a-chip platforms to study cancer-immune system crosstalk in the era of immunotherapy. Lab. Chip.

[116] Franzen N., van Harten W.H., Retèl V.P., Loskill P., van den Eijnden-van Raaij J., IJzerman M. (2019). Impact of organ-on-a-chip technology on pharmaceutical R&D costs. Drug Discov. Today.

[117] Wan L., Neumann C.A., LeDuc P.R. (2020). Tumor-on-a-chip for integrating a 3D tumor microenvironment: Chemical and mechanical factors. Lab. Chip.

[118] Haque M.R., Wessel C.R., Leary D.D., Wang C., Bhushan A., Bishehsari F. (2022). Patient-derived pancreatic cancer-on-a-chip recapitulates the tumor microenvironment. Microsyst. Nanoeng..

[119] Lai Benjamin F.L., Lu Rick X., Hu Y., Davenport H.L., Dou W., Wang E.Y., Radulovich N., Tsao M.S., Sun Y., Radisic M. (2020). Recapitulating pancreatic tumor microenvironment through synergistic use of patient organoids and organ-on-a-chip vasculature. Adv. Funct. Mater..

[120] Aleman J., Skardal A. (2019). A multi-site metastasis-on-a-chip microphysiological system for assessing metastatic preference of cancer cells. Biotechnol. Bioeng..

[121] Silvani G., Basirun C., Wu H., Mehner C., Poole K., Bradbury P., Chou J. (2021). A 3D-Bioprinted Vascularized Glioblastoma-on-a-Chip for Studying the Impact of Simulated Microgravity as a Novel Pre-Clinical Approach in Brain Tumor Therapy. Adv. Ther..

[122] Qu J., Kalyani F.S., Liu L., Cheng T., Chen L. (2021). Tumor organoids: Synergistic applications, current challenges, and future prospects in cancer therapy. Cancer Commun..

