# Supplementary material for: Global Literature Analysis of Tumor Organoid and Tumor-on-Chip Research
Source: Cancers (Basel). 2025 Jan 1;17(1):108. doi: 10.3390/cancers17010108 (PMC11719888; doi:10.3390/cancers17010108)
Supplement: Supplementary file 1 [file cancers-17-00108-s001.zip › supplementary figures.pdf]

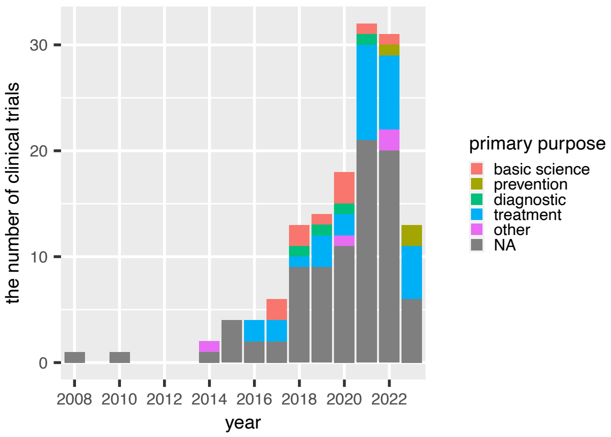

Figure S1. ***The number of clinical trials using tumor organoids and/orToC.***

Bar chart showing the yearly numbers of clinical trials registered at the ClinicalTrials.gov (https://clinicaltrials.gov) that use tumor organoid and/or ToC. The "year" data was taken from the starting date. As tumor organoids were only established in 2011, clinical trials that predate it were probably updated at a later time point to include organoid models. The bars are composed of the "primary purpose" category of the clinical trial metadata, where the "NA" means a document did not have an entry for this category.

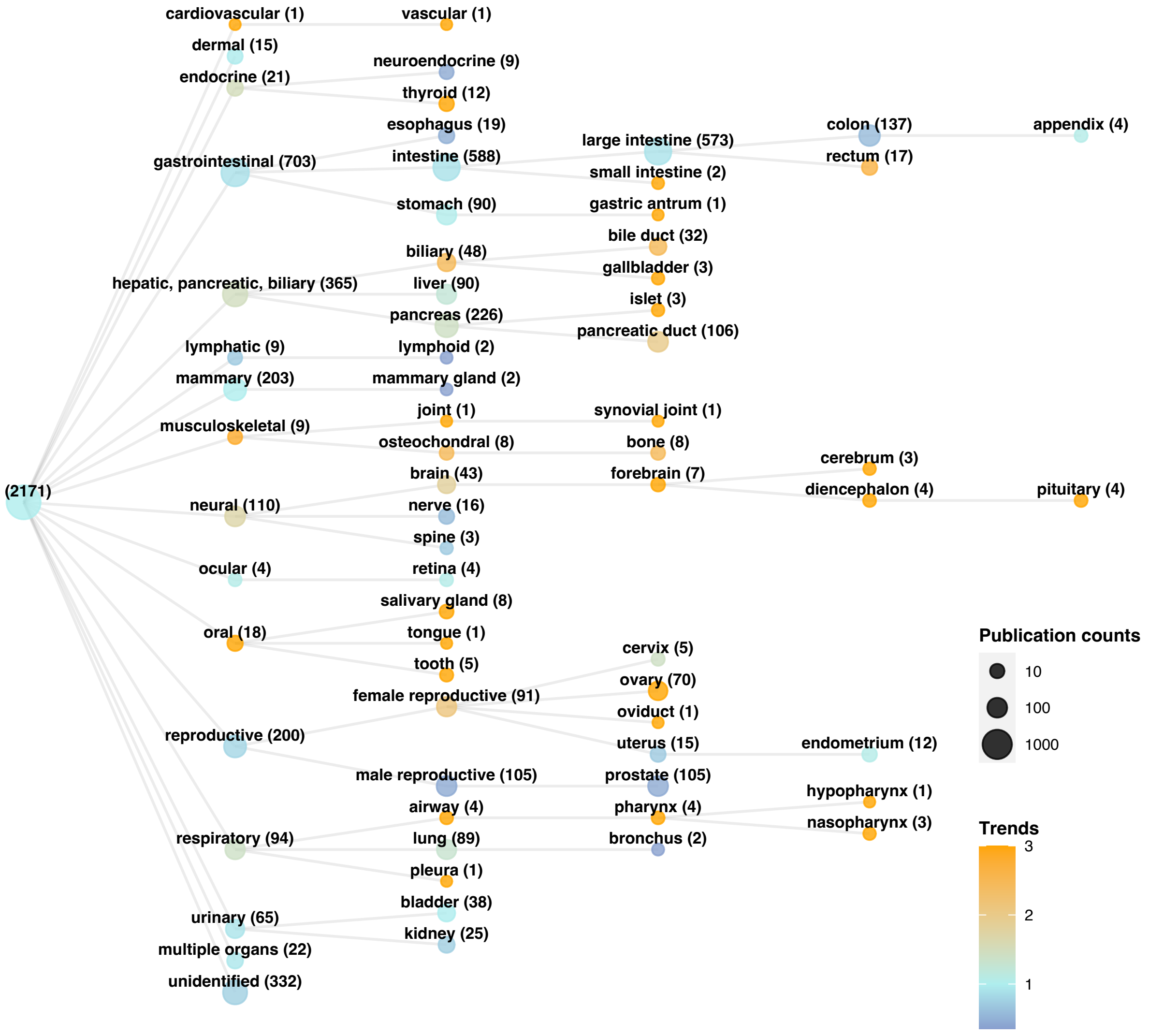

Figure S2. ***Hierarchical arrangement of organ and organ substructures modelled as tumor organoids.***

Dendrogram showing hierarchically arranged organs and organ substructures that are modelled as tumor organoids. Note that the arrangement was made to best summarize the reported organoid models and is not intended to serve as general classification of organs/substructures. Only research articles were included in the plot (2171 research articles out of 3066 academic publications). The second column from the left largely corresponds to the organ systems that were used as the major grouping of organ models throughout this paper. Note that lower-level categories merely reflect the arrangement of reported organoid models, such that a category level may encompass organ/substructures with varying histological complexity among the organ systems. Sphere size represents the number of research articles regarding the corresponding tumor organoid model, with the exact numbers shown within parentheses in the text label. The color shows publication trends as calculated by relative increases in the number of research articles in recent years (from 2020 onwards in orange) compared to the earlier period (2011 – 2019 in blue) on each organ/substructure, which were then adjusted for the relative increase in the entire corpus. Note, the "unidentified" category represents research articles without a computer-determined organ category, and was omitted from calculations in Fig 1. The "neuroendocrine" category under "endocrine" was only used when it was the only category that the algorithm detected in a publication. When another organ category was detected, the publication was categorized according to this other organ.

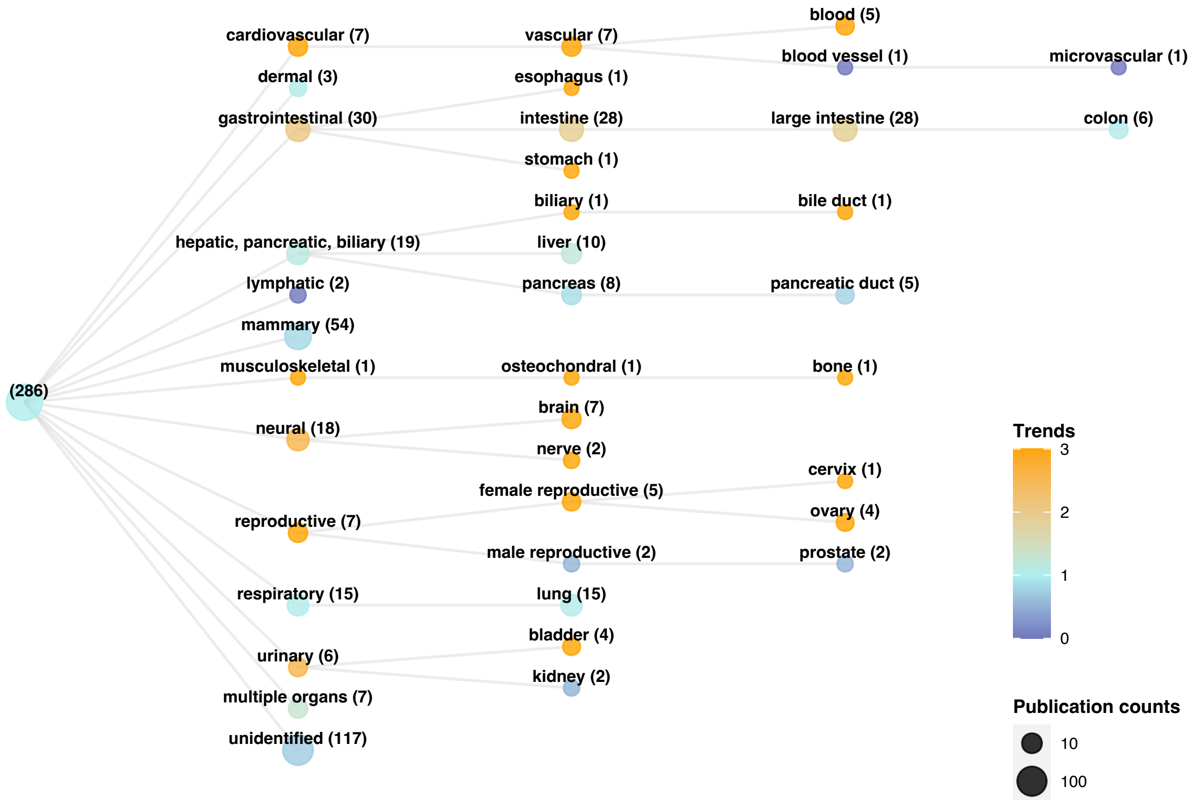

Figure S3. ***Hierarchical arrangement of organ and organ substructures modelled as ToC.***

Dendrogram showing hierarchically arranged organs and organ substructures that are modelled as ToC. Only research articles were included in the plot (286 research articles out of 485 academic publications).

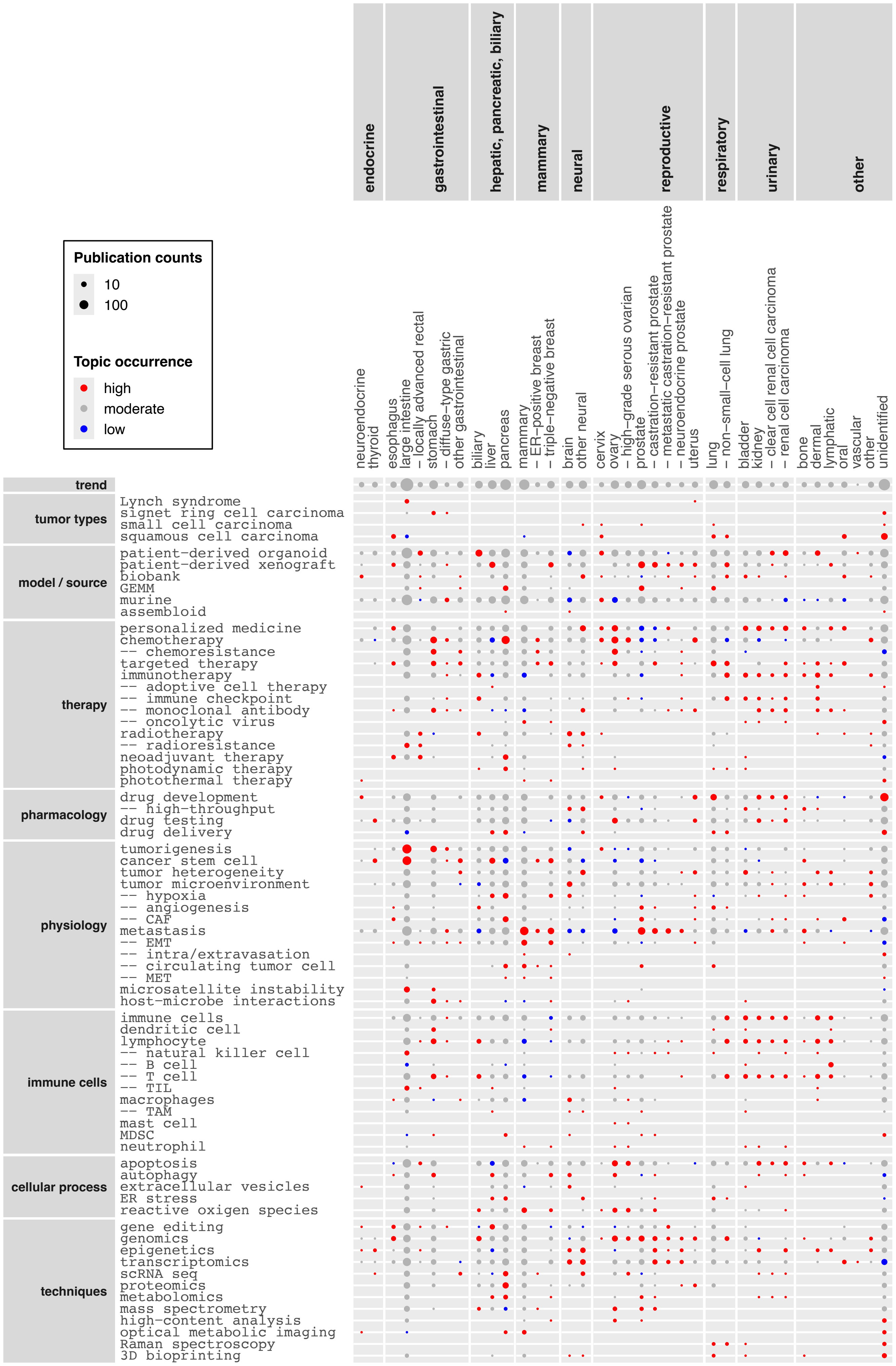

Publication counts
10
100
Topic occurrence
high
moderate
low
endocrine
gastrointestinal
hepatic, pancreatic, biliary
mammary
neural
reproductive
respiratory
urinary
other
neuroendocrine
thyroid
esophagus
large intestine
– locally advanced rectal
stomach
– diffuse–type gastric
other gastrointestinal
biliary
liver
pancreas
mammary
– ER–positive breast
– triple–negative breast
brain
other neural
cervix
ovary
– high–grade serous ovarian
prostate
– castration–resistant prostate
– metastatic castration–resistant prostate
– neuroendocrine prostate
uterus
lung
– non–small–cell lung
bladder
kidney
– clear cell renal cell carcinoma
– renal cell carcinoma
bone
dermal
lymphatic
oral
vascular
other
unidentified
trend
tumor types
Lynch syndrome
signet ring cell carcinoma
small cell carcinoma
squamous cell carcinoma
model / source
patient-derived organoid
patient-derived xenograft
biobank
GEMM
murine
assembloid
therapy
personalized medicine
chemotherapy
-- chemoresistance
targeted therapy
immunotherapy
-- adoptive cell therapy
-- immune checkpoint
-- monoclonal antibody
-- oncolytic virus
radiotherapy
-- radioresistance
neoadjuvant therapy
photodynamic therapy
photothermal therapy
pharmacology
drug development
-- high-throughput
drug testing
drug delivery
physiology
tumorigenesis
cancer stem cell
tumor heterogeneity
tumor microenvironment
-- hypoxia
-- angiogenesis
-- CAF
metastasis
-- EMT
-- intra/extravasation
-- circulating tumor cell
-- MET
microsatellite instability
host-microbe interactions
immune cells
immune cells
dendritic cell
lymphocyte
-- natural killer cell
-- B cell
-- T cell
-- TIL
macrophages
-- TAM
mast cell
MDSC
neutrophil
cellular process
apoptosis
autophagy
extracellular vesicles
ER stress
reactive oxigen species
techniques
gene editing
genomics
epigenetics
transcriptomics
scRNA seq
proteomics
metabolomics
mass spectrometry
high-content analysis
optical metabolic imaging
Raman spectroscopy
3D bioprinting

Figure S4. ***Research topic occurrences by tumor types in tumor organoid research.***

The correlation matrix shows correlation between researched tumor groups (in x-axis) and selected research topics (in y-axis), along with their relative frequency of occurrences. The size of the spheres reflects the number of research articles in the tumor groups that mention the respective research topics. The color of the spheres shows how frequently research topics are mentioned in research articles of corresponding tumor groups, relative to the average occurrences across the corpus. >40% higher occurrences were considered as "high", whereas >40% lower occurrences were considered as "low". Values in-between were classified as "moderate". The x-axis category "unidentified" encompasses research articles where the algorithm did not detect researched tumor type. The research topic categories in the y-axis represent groups of synonyms and similar terms. For example, "personalized medicine" includes precision medicine, personalised medicine, and other terms. Abbreviations in y-axis; GEMM; genetically engineered mouse model, CAF: cancer-associated fibroblast, EMT: epithelial-mesenchymal transition, MET; mesenchymal-epithelial transition, TIL: tumor-infiltrating lymphocyte, TAM: tumor-associated macrophage, MDSC; myeloid-derived suppressor cell, ER stress: endoplasmic reticulum stress, scRNA seq: single-cell RNA sequencing.
